# Bioinspired rational design of bi-material 3D printed soft-hard interfaces

**DOI:** 10.1038/s41467-023-43422-9

**Published:** 2023-12-12

**Authors:** M. C. Saldívar, E. Tay, A. Isaakidou, V. Moosabeiki, L. E. Fratila-Apachitei, E. L. Doubrovski, M. J. Mirzaali, A. A. Zadpoor

**Affiliations:** 1 Department of Biomechanical Engineering, Faculty of Mechanical, Maritime, and Materials Engineering, Delft University of Technology (TU Delft), Mekelweg 2, 2628 CD Delft, The Netherlands; 2 Faculty of Industrial Design Engineering (IDE), Delft University of Technology (TU Delft), Landbergstraat, 15, 2628 CE Delft, The Netherlands

**Keywords:** Polymers, Composites, Design, synthesis and processing

## Abstract

Durable interfacing of hard and soft materials is a major design challenge caused by the ensuing stress concentrations. In nature, soft-hard interfaces exhibit remarkable mechanical performance, with failures rarely happening at the interface. Here, we mimic the strategies observed in nature to design efficient soft-hard interfaces. We base our geometrical designs on triply periodic minimal surfaces (i.e., Octo, Diamond, and Gyroid), collagen-like triple helices, and randomly distributed particles. A combination of computational simulations and experimental techniques, including uniaxial tensile and quad-lap shear tests, are used to characterize the mechanical performance of the interfaces. Our analyses suggest that smooth interdigitated connections, compliant gradient transitions, and either decreasing or constraining strain concentrations lead to simultaneously strong and tough interfaces. We generate additional interfaces where the abovementioned toughening mechanisms work synergistically to create soft-hard interfaces with strengths approaching the upper achievable limit and enhancing toughness values by 50%, as compared to the control group.

## Abstract (web summary)

Stress concentrations make the design of durable interfaces between hard and soft materials a challenging task. Here, the authors, inspired by nature, combine mechanical tests and simulations on multiple geometries to generate design guidelines that yield strong and tough soft-hard interfaces.

## Introduction

Joining materials with dissimilar mechanical properties is inherently challenging due to the complexities present at the soft–hard interfaces^1–3^. These complexities include the different load-carrying capacities of both materials, interfacial damages caused by the failure of any adhesives present at the interface, and the stress concentrations caused by the sudden changes in the material properties^4–7^. Among those factors, the lattermost is particularly concerning because interfacial microarchitecture and geometry play key roles in the development of stress singularities^8^. In contrast, several millennia of evolution endow naturally architected structures with remarkable mechanical properties that originate from their complex yet highly efficient arrangements of mechanically dissimilar phases^9–11^. Given the failure of engineered constructs in reproducing the high level of efficiency exhibited by natural materials, it is important to understand and mimic the naturally occurring design strategies.

A prime example of such a high-performing interface is the tendon enthesis, where the soft tendon connects to the much stiffer bony tissue along a relatively short transitional length^12^, employing an efficient mixture of design features, such as morphological interdigitations and anisotropic orientations^13–16^. Moreover, functional gradients (FGs) enable a smooth transition of material properties from bone to tendon, reducing interfacial stresses^17–21^. The synergy of these mechanisms makes the bone–tendon connection highly efficient^12^.

To date, a major impediment to the application of such design features has been the lack of suitable manufacturing techniques. The emergence of multi-material additive manufacturing (=3D printing) techniques has addressed this limitation and has enabled us to closely emulate the abovementioned natural design paradigms. These techniques allow for the design of structures with interpenetrating soft and hard material phases, yielding composites with optimized properties^22–26^. In particular, controlling the type of the deposited material at the level of individual voxels makes Polyjet multi-material 3D printing highly suitable for the emulation of natural soft–hard interfaces^27–29^.

Many types of architectures could be used as a basis for the design of biomimetic interfaces, particularly those that involve complex 3D architectures. However, most of the designs available in the literature are 2D and, at best, 2.5D^14^. This limitation leaves much of the potential of geometrical designs unexploited. Here, we select a few types of architectures to study the effects of architecture types and design parameters on the mechanical performance of the resulting soft–hard interfaces. Triply periodic minimal surfaces (TPMS)^30–32^ are selected architectures because they offer large surface area to volume ratios and high genus values, both of which are highly beneficial for an enhanced interlocking of the interfacing phases. A high genus value means that there are multiple surface-connected yet volume-separated compartments available in the architecture of the material. Each of those compartments could be occupied by one of the interfacing phases. In this way, the phases interlock volumetrically across the vast surface area of the unit cells. A higher contact area between the material phases can also reduce strain concentrations. Architectures based on collagen-like helices^33,34^ are also considered because they facilitate the creation of functional gradients while offering open cells and high surface areas. The design matrix is complemented by including randomly distributed particles, which are known to generate smooth functional gradients and arrest propagating cracks^35^. Moreover, the random distribution of particles can be integrated into multi-hierarchical arrangements^36^ to prevent failure within the interface region.

In this study, we use both experiments and computational models to compare the various design options mentioned above and to elucidate the mechanisms determining the relative performance of different architectures. The experiments, which include uniaxial tensile and quad-lap shear tests as well as their associated full-field strain measurements using digital image correlation (DIC), allow for a comprehensive and multi-faceted evaluation of the mechanical performance of soft-hard interfaces. At the same time, our computational models enable a thorough analysis of the mechanistic aspects driving the performance of such interfaces, including the role of the incorporated 3D geometrical design features. In particular, we study the relation between the internal geometry, the type of the transition function, and the contact surface on the one hand and the mechanical characteristics of the soft–hard interfaces, and the ensuing strain concentrations^37^ on the other. This approach provides us with a pathway towards a better understanding of the mechanisms at play in the design of soft–hard interfaces and enables us to devise some design guidelines for improving the mechanical performance of bioinspired soft–hard interfaces, with potential applications in tissue engineering, soft robotics, and architected materials.

## Results and discussion

**Fig. 1:**
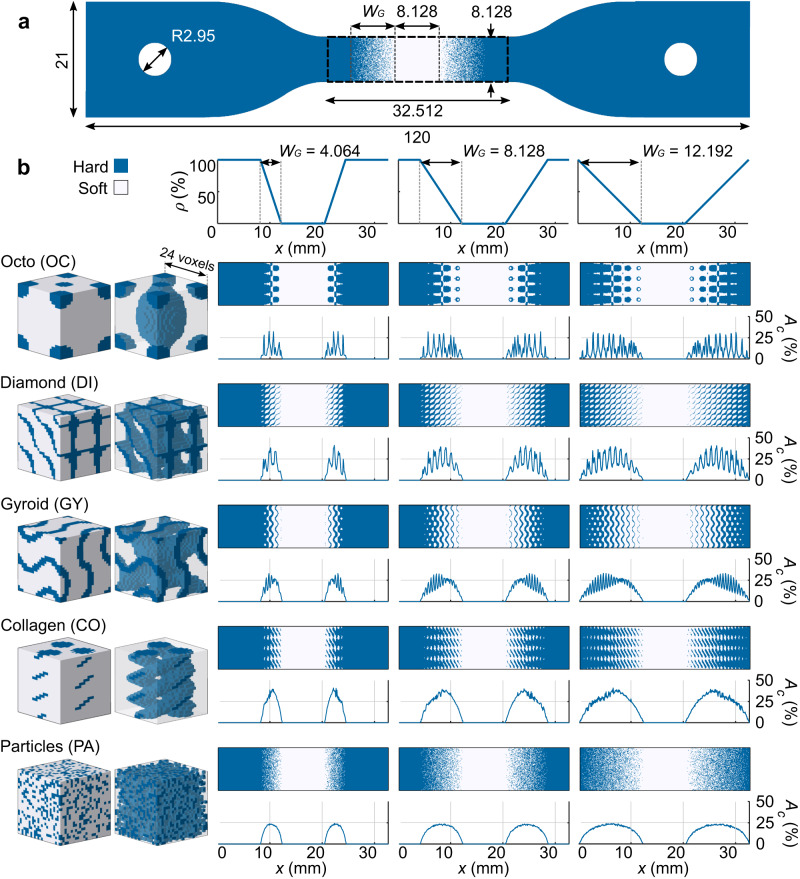
The soft–hard interface designs tested under tensile conditions. **a** The standard tensile test specimens furnished with a functional gradient connecting the hard and soft polymer phases through linear functions of hard phase volume fraction ($\rho$) (out-of-plane thickness = 4 mm). These designs were 3D printed using a Polyjet multi-material 3D printer. **b** All the initial designs with different functional gradient widths (${W}_{\rm {{G}}}$) and their calculated percentage of the soft–hard normal contact area (${A}_{\rm {{c}}}$). We combined three different values of the gradient length (${W}_{\rm {{G}}}$) with five different unit cell geometries (i.e., Octo, Diamonds, Gyroids, collagen-like helices, and randomly distributed particles).

**Fig. 2:**
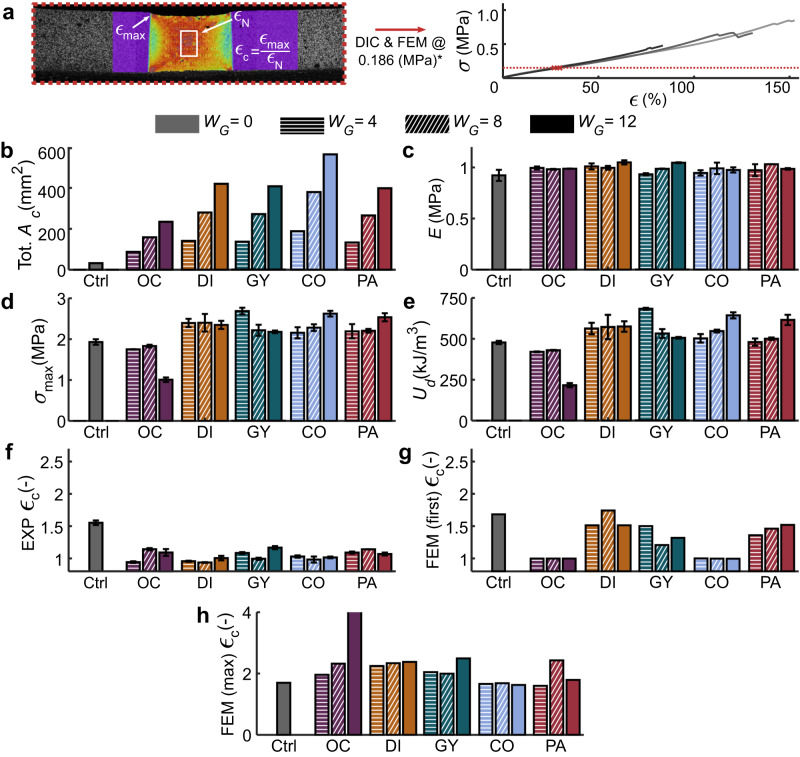
The overall mechanical performance of the soft–hard interfaces tested under tensile conditions. **a** A representative example of a DIC measurement showing the regions from which the strain concentration parameters (${\epsilon }_{\rm {{c}}}$) were calculated (obtained at the point where the equivalent stresses were 0.1866 MPa). The bar plots represent the average morphological and mechanical properties of the studied designs (with error bars representing ±SD). These include **b** the total soft–hard normal contact area (${{{{{\rm{Tot}}}}}}.\,{A}_{{\rm {C}}}$), **c** measured elastic modulus (*E*), **d** maximum strength (${\sigma }_{\max }$), **e** strain energy density (${U}_{{{{{{\rm{d}}}}}}}$), **f** experimentally (EXP) measured ${\epsilon }_{\rm {{c}}}$, and the (FEM) predicted ${\epsilon }_{\rm {{c}}}$ results obtained from the top (i.e., first) layer of the meshes (**g**) and the maximum value within from the entire (max) 3D structure (**h**).

The integration of the different architectures into the soft-hard interfaces led to distinct patterns of percentage of soft–hard normal contact area (${A}_{\rm {{c}}}$) (Fig. 1 and Supplementary Fig. 1a) and resulted in different total values of the contact surface area (${{{{{\rm{Tot}}}}}}.{A}_{\rm {{c}}}$) (Fig. 2b). None of these differences had a considerable effect on the initial elastic moduli (Fig. 2c) calculated using the obtained stress–strain curves (Supplementary Fig. 2). Moreover, the behavior of all the stress-strain curves was similar, where a nonlinear (i.e., hyperelastic) increase of stress was present until sudden failure at high strain values (e.g., ultimate strain >80%) with little to no strain softening prior to failure, indicating that little to no plastic behavior was present for the soft–hard interfaces. Nevertheless, the varying geometries and gradient lengths did affect the strength and toughness of the interfaces (Fig. 2d, e). The best-performing designs were the Gyroid (GY, with the functional gradient width of ${W}_{\rm {{G}}}=4{{{{{\rm{mm}}}}}}$), Collagen (CO, ${W}_{\rm {{G}}}=12\,{{{{{\rm{mm}}}}}}$), and Particles (PA, ${W}_{\rm {{G}}}=12\,{{{{{\rm{mm}}}}}}$). They all exhibited similar strengths and failure modes (i.e., failure within the soft region), suggesting that the upper strength boundary of these interfaces was reached^38^. The control group under-performed all but the Octo (OC) designs, confirming the importance of the implemented design strategies in improving the mechanical performance of soft–hard interfaces.

**Fig. 3:**
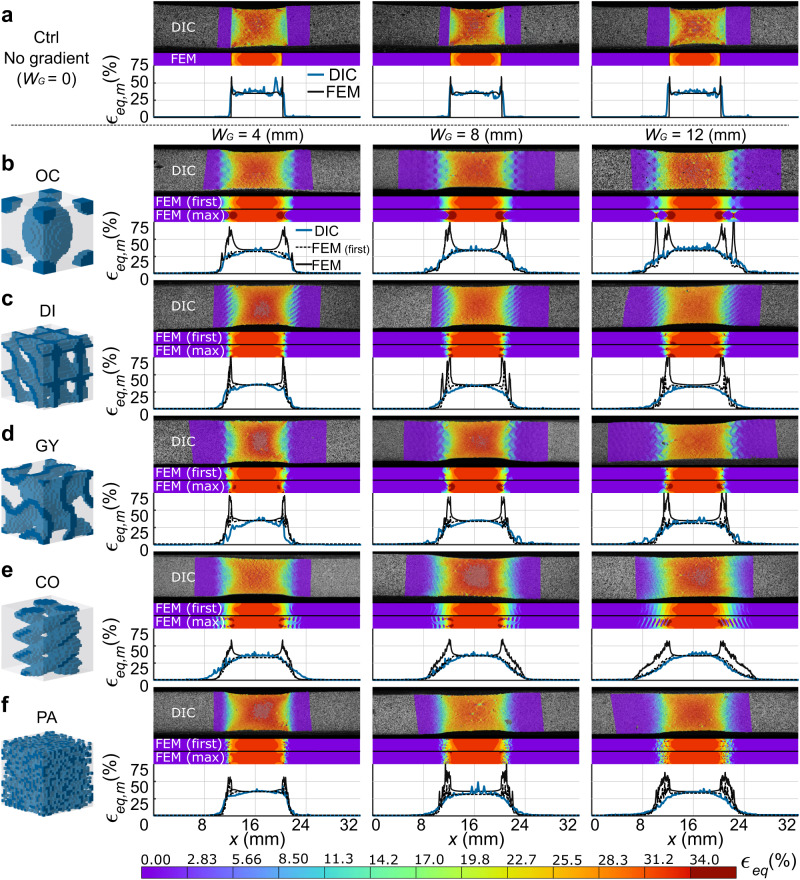
Representative strain distributions measured using DIC and predicted using the FEM models as well as the maximum equivalent strain (*ϵ*_eq,m_) plots for every design. The study groups include the **a** control group, **b** Octo, **c** Diamond, **d** Gyroid, **e** collagen-like helices, and **f** randomly distributed particles. The FEM strain distributions are presented for the first layer and at the location where the maximum strains were predicted, for validation and analysis purposes, respectively.

We found some evidence of the mechanism responsible for the failure of the control group specimens when assessing their strain concentration parameter (${{{{{\rm{EXP}}}}}}\,{\epsilon }_{\rm {{c}}}$) values (Fig. 2f) and strain distributions measured with digital image correlation (DIC) (Fig. 3a). According to this data, the shear strains at the edges of the interface were the primary culprits. This observation as well as the absence of high values of the von Mises strains (${\epsilon }_{{\rm {{eq}}}}$) at the center of the cross-section cut of the interface are consistent with the literature^4,39,40^. These superficial strain concentrations were not present in the DIC results of any functionally graded design. Moreover, the ${{{{{\rm{EXP}}}}}}\,{\epsilon }_{\rm {{c}}}$ values of the other groups were all much lower than those measured in the control group. This lack of shear strains explains the improved performance of most of the presented designs because proper interfacing of soft and hard materials requires a smooth transition from one phase to another so that the stress concentration in the softer material can be decreased^41^. The difference between the FEM-predicted (Supplementary Fig. 3) ${\epsilon }_{\rm {{c}}}$ values pertaining to the first layer of the models and those of the entire 3D structure (Fig. 2g–f) indicates that the strain concentrations occurring within the 3D structure of the constructs may not always be fully visible on the surface. Therefore, a closer inspection of the results of the FEM simulations was necessary to elucidate the effects of the gradient morphology on the mechanical performance.

Comparing the DIC-measured strain distributions with the FEM results extracted from the first layer of the meshes allowed us to validate our computational models (Fig. 3b–f). In general, the predicted and measured strain distributions followed the same patterns and were strongly correlated (*R*^2^ > 92.1%) (Supplementary Fig. 4). For example, all the GY designs showed curved-like strain patterns in both the experiments and simulations. In contrast, the strain distribution patterns of the Diamond (DI) and CO designs presented distinct diagonal lines. Similarly, the trends observed in the FEM-obtained maximum equivalent strains (${\epsilon }_{{\rm {{eq},m}}}$) plots (of the first layer) resembled the DIC measurements. The simulations, however, showed higher peak strain values as compared to the experiments (e.g., see the peaks at the edges of the DI designs or between individual voxels in the PA gradients). The absence of these peaks was likely caused by the limited DIC resolution (i.e., between 22 × 10^3^ and 27 × 10^3^ facets per experiment), which was approximately six times lower than the resolution of the 3D-printed specimens (i.e., 147 × 10^3^ voxels). Furthermore, blending of the photopolymers prior to curing might have significantly reduced the magnitudes of the strain peaks^42,43^. The FEM predictions were, therefore, more discerning when trying to understand the effects of geometrical design on the mechanical performance of soft–hard interfaces. An additional set of simulations was, however, necessary to assess if simulating only a single unit cell instead of the entire interface was representative of the complete interfaces (Supplementary Note 2 and Supplementary Fig. 5). This comparative study demonstrated that, while some differences are present in terms of the absolute strain values, the overall mechanical behavior, the deformation trends, and the elastic modulus functions ($E(x)$) remain consistent between the single unit cell models and models incorporating the actual full-size geometry of the specimens. Moreover, an additional analysis of the 3D behavior of interfaces under loading demonstrated that the overall behavior of graded (i.e., long OC and PA) and non-graded specimens measured with a micro-CT scanner followed the same trends as those estimated with the FEM simulations (Supplementary Note 3 and Supplementary Fig. 6). This was a welcome outcome because it enabled us to use the single-unit cell models in the remainder of the study to evaluate the performance of a large number of design alternatives. This corroboration of the computational results allowed us to analyze the different designs individually in a quest to unravel the mechanisms underlying their mechanical performance.

Although generally better than the control group, most OC and DI specimens failed at the edge of the interface (only one DI specimen $({W}_{\rm {{G}}}=8\,{{{{{\rm{mm}}}}}})$ failed at the center of the soft region). An analysis of the FEM-predicted strain distributions of these groups showed the prevalence of severe strain concentrations at their interface edges (Fig. 3b, c, Supplementary Videos 1 and 2), whose intensity was correlated to ${W}_{\rm {{G}}}$. Upon closer inspection (Supplementary Fig. 7b, c), the sharp-edged tips of the hard material at the edge of the interface seemed to have induced these strain concentrations. These shapes are particularly problematic since the interfacial geometry cannot arrest the propagation of initial cracks. Comparing the ${A}_{\rm {{c}}}$ and ${\epsilon }_{\rm {{{eq},m}}}$ plots (Supplementary Fig. 8a, b) of both designs indicated that these strain concentrations are associated with highly erratic ${A}_{\rm {{c}}}$ patterns. A smooth material transition may, thus, alleviate such effects. It is, therefore, necessary to change the density of the hard phase ($\rho$) as smoothly as possible by using a geometry for which the change in ${A}_{{\rm {c}}}$ is less abrupt, and ensure that there are no sharp ends in the selected geometrical design.

In the case of the long OC design (i.e., ${W}_{{\rm {G}}}=12\,{{{{{\rm{mm}}}}}}$), the hard material discontinuity at the middle of the interface resulted in extreme strain concentrations (${{{{{\rm{FEM}}}}}}(\max ){\epsilon }_{{\rm {c}}}=4.53$) and was the region where critical cracks were initiated. This lack of connectivity, which is visible in the discontinuous ${A}_{\rm {{c}}}$ pattern of this design and in Supplementary Video 1, led to extremely low values of interfacial strength and toughness (i.e., approximately half the strength and toughness of the control group, Supplementary Table 1). Therefore, although TPMS structures can yield closed-cell structures, verifying their connectivity by assessing their surface contact area and making the necessary corrections to $\rho$ is of great importance.

The GY results were particularly interesting because long gradients from this design (${W}_{{\rm {G}}}=12\,{{{{{\rm{mm}}}}}}$) barely overperformed the control group, while the shorter version of the same design (${W}_{{\rm {G}}}=4\,{{{{{\rm{mm}}}}}}$) outperformed all the other groups (Fig. 2d, e). The short GY gradients presented failure modes where cracks initiated close to the interface but propagated through the soft region of the tensile specimens (Supplementary Video 3). In contrast, the other GY interfaces failed at the end of the interface. This performance difference can be due to several reasons. First, the GY specimens had ${A}_{\rm {{c}}}$ patterns that were not as torturous as the OC and DI designs (Supplementary Fig. 8c), explaining why their predicted ${{{{{\rm{FEM}}}}}}\,{\epsilon }_{\rm {{c}}}$ values were the lowest between the TPMS structures. More importantly, the strains of the short GY gradients mainly concentrated around the concave hard material shapes before the end of the gradient and at the edges of the interface, with the maximum strain values appearing close to the sheet-based Gyroid geometry (Supplementary Fig. 7d). This concave geometry, in turn, encased the regions with maximum strain concentrations, arresting the critical propagation of cracks. In comparison, the longer GY gradients mostly showed tip-edged strain concentrations at the soft ends of the interface, similar to those found in the OC and DI designs. Although the smooth ${A}_{\rm {{c}}}$ pattern appears to have contributed to the high performance of short GY designs, comparison with the other designs indicates that the ability to contain the strain concentrations may have played a more important role in this regard, particularly given the fact that the short GY design was the only studied TPMS with this feature.

An important parameter affecting the performance of the soft-hard interface is the length over which the transition takes place (i.e., ${W}_{\rm {{G}}}$). The performance of a soft–hard interface is generally expected to improve as the length of the gradient increases, given that longer transitions lead to smoother changes in the elastic modulus, decreasing stress concentrations^41^. Indeed, the plots of the elastic modulus of the TPMS designs (Supplementary Fig. 8a–c) were increasingly smoother as ${W}_{{\rm {G}}}$ increased. The performance of the TPMS designs was, however, inversely related to ${W}_{{\rm {G}}}$, as the strength values were higher for the specimens with shorter gradients while the ${{{{{\rm{FEM}}}}}}\,{\epsilon }_{\rm {{c}}}$ values were higher for the longer specimens. The local geometrical features at the end of a functional gradient are, therefore, more important in determining the mechanical performance of the interface than the overall smoothness of the function describing the transition of the elastic modulus. Consequently, it is important to utilize geometries that reduce the strain concentrations as ${W}_{{\rm {G}}}$ increases or to include features that help in arresting cracks.

In contrast with the TPMS structures, the mechanical performance of the CO and PA designs was enhanced as ${W}_{{\rm {G}}}$ increased. In fact, the CO and PA designs with long gradients (${W}_{{\rm {G}}}=12\,{{{{{\rm{mm}}}}}}$) were some of the toughest designs within this study (Supplementary Videos 4 and 5). Moreover, most specimens of these two groups failed at the center of the soft region and not at the interface. When analyzing the FEM-predicted strain distributions of the CO designs (Fig. 3d, Supplementary Fig. 7e), we observed that the deformations were primarily concentrated in the soft material regions between each coil of the functional gradient, with the cross-sectional strains showing circular patterns of strain that were reminiscent of helices. These patterns resulted in smooth ${\epsilon }_{{\rm {{eq},m}}}$ plots (Supplementary Fig. 8d) and the lowest FEM ${\epsilon }_{\rm {{c}}}$ values of this study. Such a proper distribution of strains may be attributed to the high ${{{{{\rm{Tot}}}}}}.\,{A}_{\rm {{c}}}$ values and smooth ${A}_{\rm {{c}}}$ patterns of these designs, which are similar to what is reported in the literature^39^. Furthermore, their elastic modulus functions were the most compliant, explaining the presence of strains across the longer sections of the gradient region (unlike in the TPMS results, where the strains were concentrated at the edges). Particularly for the long CO design, the smooth and well-distributed strains across the entire gradient region indicated an increased strain energy storage capacity, leading to high toughness values.

For the PA designs, the predicted ${{{{{\rm{FEM}}}}}}\,{\epsilon }_{\rm {{c}}}$ values were not proportional to ${W}_{\rm {{G}}}$ and the strain distributions exhibited irregular patterns that resembled the random nature of the designs (Fig. 3f). The locations of these strain concentrations were not necessarily at the end of the interface, but in the single voxels of the soft material surrounded by the hard material across the entire gradient (Supplementary Fig. 7f). If a crack initiates around those stress concentration points, the nearby voxels could deflect it or arrest its progress, similar to what other studies have observed^44^. Furthermore, the comparatively higher magnitudes of strains across the gradient length produced more compliant elastic modulus functions in these designs, similar to the CO specimens (Supplementary Fig. 8e). These high strains mean that more strain energy is stored in such specimens, resulting in higher toughness values, particularly for the long PA gradients. Overall, both CO and PA achieved their high toughness because they could store more energy in their gradient region and because of the crack-arresting features of their internal morphology.

**Fig. 4:**
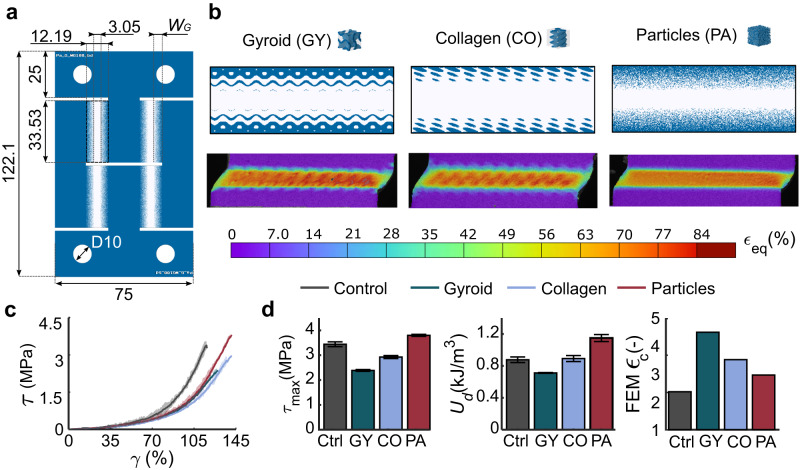
The specimens used in the shear tests and their corresponding results. **a** The parametrized geometry of the quad-lap shear test specimens, with an out-of-plane thickness of $3\,{{{{{\rm{mm}}}}}}$ and ${W}_{{\rm {G}}}=3.048\,{{{{{\rm{mm}}}}}}$. **b** The selected geometries (e.g., GY, CO, and PA), chosen due to their high tensile performance. The presented DIC measurements of the equivalent shear strains correspond to a shear stress of $0.523\,{{{{{\rm{MPa}}}}}}$. **c** The average (with shaded areas representing ±SD) shear stress vs. shear strain ($\tau$ vs. $\gamma$) curves for every design. **d** The bar plots represent the mean measured shear modulus ($G$), strain energy density (${U}_{{\rm {d}}}$), and estimated FEM ${\epsilon }_{\rm {{c}}}$ for each design (with error bars representing ±SD).

We have so far only considered tension because this loading mode is typical in soft-hard interfaces (e.g., cables, tendons, muscles). Soft–hard interfaces may, however, also fail under shear deformations, motivating the study of the presented designs under this loading regime. Since no standards are available for the geometrical design of functionally graded quad-lap specimens, a custom-made design was used (Fig. 4a), enabling us to manufacture the specimens and perform mechanical testing (Fig. 4b, Supplementary Videos 6–8). After post-processing, all the hyperelastic shear stress–strain ($\tau -\gamma$) curves had similar initial values of the shear modulus (${G}_{{{{{{\rm{avg}}}}}}}=0.308\,{{{{{\rm{MPa}}}}}}$, Fig. 4c), confirming the proper design of the specimens and the satisfactory distribution of materials in the presented designs. Moreover, the $\tau -\gamma$ curves of the control group allowed for corroboration of the fact that the selected parameters of the hyperelastic Ogden model work adequately in both uniaxial tensile and shear loading conditions (Supplementary Fig. 9). In terms of the shear strength and toughness, however, the PA designs outperformed all other groups (Fig. 4d). Moreover, the performance of the specimens was inversely related to their predicted FEM ${\epsilon }_{\rm {{c}}}$, suggesting that the internal morphology of these interfaces was responsible for this outcome. Based on these observations, we concluded that using PA reinforcement in the design of soft–hard interfaces can enhance their performance under shear deformations.

**Fig. 5:**
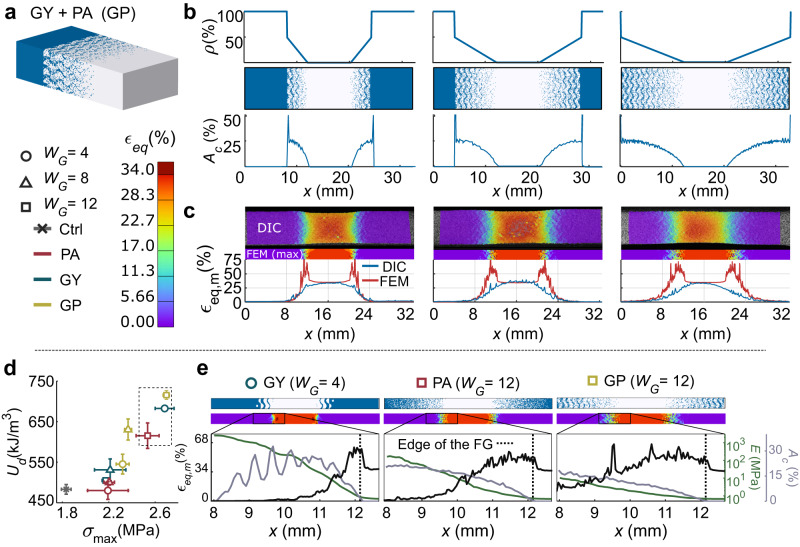
The results of the hybrid designs. **a** These designs were obtained by combining Gyroid geometries with randomly distributed particles (GP) through a multi-scale approach. **b** The magnitude of the $\rho$ functions were decreased to produce more compliant functional gradients while maintaining smooth ${A}_{\rm {{c}}}$ patterns. **c** Representative strain distributions measured using DIC and predicted using computational models as well as ${\epsilon }_{{\rm {{eq},m}}}$ plots for these designs. **d** A scatterplot comparing the ${\sigma }_{\max }$ vs. ${U}_{{\rm {d}}}$ results for the GY, PA, and GP designs (with error bars representing ±SD). The GP specimens were among the best-performing ones. **e** A detailed comparison of the mechanistic features of the best-performing GY, PA, and GP designs.

In summary, the data and analysis presented above indicate that there are several morphological and mechanical principles that can lead to a tough soft–hard interface. First, the ${A}_{{\rm {c}}}$ must be smooth to prevent any sudden changes in ${\epsilon }_{{\rm {{eq},m}}}$ across the functional gradient. Second, the corresponding ${{{{{\rm{Tot}}}}}}.\,{A}_{\rm {{c}}}$ values should be as high as possible to decrease the overall magnitude of strain concentrations. Furthermore, it is important for the functional gradients to be increasingly more compliant, particularly at the edges of the interface. This compliance will lead to increased average deformations across the entire interface length. Higher amounts of strain energy can, therefore, be stored in the gradient region, leading to diffused stress concentrations and tougher soft–hard interfaces. Finally, the design of the structures should be such that any initiated cracks can be arrested, particularly at the end of the gradient region. It is, therefore, essential to include analyses of the entire 3D geometry of the interface across every cross-section. Examples of such geometries include concave designs implemented around strain concentration regions and randomly distributed particles. The selected geometries should also not include sharp tips of the hard phase at the edges of the interface because they create strain concentrations. Since most of the studied designs failed to implement all the aforementioned morphological features, we decided to extend our analysis by creating a design that combines the best-performing TPMS (i.e., the GY) with PA (Fig. 5a, b). We hypothesized that adding particles to a Gyroid design (GY + PA = GP) will hinder the propagation of critical cracks while producing a smooth ${A}_{\rm {{c}}}$ pattern. Additionally, we decreased the ratio of the hard material, $\rho$, to 50% of its original value so that higher magnitudes of strain energy could be accommodated by the interface.

Our experiments confirmed that the GP specimens, indeed, exhibit many of the characteristics of high-performing soft–hard interfaces. The DIC-measured strain distributions of the GP specimens (Fig. 5c, Supplementary Video 9) shared certain features with both the GY and PA designs while lacking any significant superficial strain concentrations. Overall, the 3D FEM models showed higher strain magnitudes and relatively higher levels of strain concentrations across the length of the functional gradient (Supplementary Fig. 7g). All in all, the GP designs yielded the smoothest modulus functions in this study, enabling more strain energy to be stored within the FG. Furthermore, their ${\epsilon }_{{\rm {{eq},m}}}$ plots showed that the strain concentrations vanish before the end of the gradient region (Supplementary Fig. 8f). Similar to the short GY and long PA designs, the presence of hard material around the locations of peak strains indicates that the interface can arrest initial cracks (Fig. 5e). The performance of the GP specimens increased with ${W}_{\rm {{G}}}$ (Fig. 5d). In fact, the long (${W}_{\rm {{G}}}=12\,{{{{{\rm{mm}}}}}}$) GP specimens presented the highest toughness values in this study (i.e., 1.48 times tougher than the control specimens). Moreover, additional ductile failure simulations showed that the failure modes and the mechanistic principles behind the performance of the GP and other geometries remained consistent with the aforementioned interface design guidelines (Supplementary Note 4, Supplementary Fig. 10, and Supplementary Videos 10–14). These results confirm that the implementation of multi-scale features into a highly interdigitated and compliant functionally graded design further enhances the performance of soft–hard interfaces.

The results obtained here are not necessarily limited to the applied multi-material Polyjet printing technique and the specific materials used here. Indeed, the focus of the current study has been on identifying the guiding principles for the (geometrical) design of soft–hard interfaces at the individual voxel level. Currently, Polyjet multi-printing is the only widely available technique that can be controlled at the individual voxel level and can process multiple material phases with vastly different mechanical properties at a sub-50 μm scale. That is why we used this technique in the current study. However, the guiding design principles and their action mechanisms are primarily geometrical in nature and are formulated in reference to the patterns of the arrangement of both material phases. That is why we expect these mechanisms to be valid for other types of material combinations and (additive) manufacturing techniques. Indeed, additional computational analyses performed using our models showed that the main guiding principles remain valid regardless of the ratio of the elastic properties of both phases (Supplementary Note 5 and Supplementary Fig. 11). Moreover, an analysis of the effects of photopolymer blending (Supplementary Note 6, Supplementary Fig. 12, and Supplementary Videos 15–18) showed that even though material mixing can improve the performance of photopolymer composites, the general observations regarding the effects of various geometrical design alternatives on the performance of the interfaces remain unchanged^42,43^. Experimental studies of such effects are currently not possible outside multi-material Polyjet printing due to technological limitations. However, it is expected that the future developments of additive manufacturing techniques and materials will enable multi-material voxel-by-voxel printing also for other types of material connections, such as metal–metal (e.g., Mg–NiTi), hydrogel–metal, and for other polymer–polymer connections processed using other additive manufacturing techniques (e.g., FDM polymers). Furthermore, additional analyses of the fracture behavior of soft–hard interfaces (Supplementary Note 7 and Supplementary Fig. 13) are generally consistent with the results of other experiments and simulations presented in the main text (i.e., tensile and shear tests) and highlight the importance of developing suitable standards for evaluating the fracture behavior of soft–hard interfaces with prescribed flaws, as well as showcasing an alternative computational method in which flaws are permuted along the FG interface to study their performance under fracture loading conditions. At its roots, this study concerns the mechanical behavior observed at the interface of two interpenetrated phases, similar to the ones found in the interdigitated functional gradients of natural tissues. Histological studies in different organisms show similar general principles at work in multiple species, various material combinations, and anatomical sites^18,45^. This further supports the idea that the main guiding principles are valid regardless of the specific material combinations or mechanical functionalities. Thus, the results obtained within this work open the door to a wide range of future studies. Examples include performing extensive computational analysis and obtaining geometries with minimized strain concentrations (e.g., through genetic algorithms), modeling such geometries through artificial neural networks, characterizing the fatigue behavior and fracture crack propagation of soft–hard interfaces, performing in-situ analyses of interfaces within a micro-CT chamber and conducting digital volume correlation measurements, analyzing 3D interfaces under multi-modal loading conditions resembling physiological equivalents, and extending the voxel-by-voxel technique to other 3D printing methods, such as metal-to-metal printing or hydrogel–metal printing. While many of these research directions require additional methodological development, their application may be motivated by the results, mechanisms, and methods presented here.

We studied how the design of soft–hard interfaces influences their mechanical performance. Our results clearly show the role of increased contact area, elastic modulus functions, and design features that attenuate or constrain strain concentrations in the rational design of high-performing soft–hard interfaces. The application of the abovementioned design features yielded soft–hard interfaces whose strength approached the upper boundary of the possible strengths and whose toughness increased by ≈50% as compared to that of the control specimens. Future work should employ these guidelines with computational methods to design optimized soft–hard interfaces. However, validating the presented design guidelines for other additive manufacturing methods that enable voxel-by-voxel deposition is imperative to overcome the limitations imposed by the properties and behavior of commercial Polyjet photopolymer materials. The presented results ultimately contribute to the development of the next generation of designer materials with applications in, among other areas, medical devices, tissue engineering, soft robotics, and the design of architected flexural mechanisms.

## Methods

### 3D printing setup

We used a Polyjet multi-material 3D printer (ObjetJ735 Connex3, Stratasys® Ltd., USA) with voxel-level control to manufacture our biomimetic soft–hard interfaces. The commercially available photopolymers VeroCyan^TM^ (RGD841, Stratasys® Ltd., USA) and Agilus30^TM^ Clear (FLX935, Stratasys® Ltd., USA) were used for the hard and soft phases, respectively. Stacks of binary images detailing the type of the deposited material at each voxel were provided as input to the printer. In this approach, every image represents a layer of the 3D design. The maximum printing resolution was 300 × 600 × 900 dpi. We used the minimum edge size to print cube-shaped voxels with an edge length of 85 ${{{{{\rm{\mu }}}}}}{{{{{\rm{m}}}}}}$.

### Design and manufacturing of tensile test interfaces

Due to the lack of standards available for the analysis of the complex 3D geometries required to analyze soft–hard interfaces, we considered the narrow section of a standard tensile test specimen shape (type IV) described in ASTM D638-14^46^ to constrain the dimensions of our designs (Fig. 1a). These dimensions allowed for a design region of 384 × 96 × 48 voxels (32.51 × 8.1 × 4.0 ${{{{{\rm{m}}}}}}{{{{{{\rm{m}}}}}}}^{3}$). Within these regions, we kept the length of the soft region constant (${W}_{\rm {{S}}}=8.128\,{{{{{\rm{mm}}}}}}$) and varied two interface parameters, namely the width and geometrical design of the functional gradient (Fig. 1b). We selected three different values of ${W}_{\rm {{G}}}$ (i.e., 4.064, 8.128, 12.192 mm, equivalent to 48, 96, and 144 voxels), wherein we linearly varied the volume fraction of the hard phase ($\rho$) from 0% to 100%. We discretized these functions using cubic-shaped unit cells with five different geometries, including three TPMS-based architectures (i.e., Octo, Diamond, and Gyroid), biomimetic collagen-like triple helices, and randomly distributed particles (Fig. 1b). Supplementary Note 1 provides the details of the equations used for the generation of each design. It is important to note that the selected discretization strategy results in a significant discontinuity in the hard phase for the long OC design (i.e., ${W}_{\rm {{G}}}$ = 12 mm). We, nevertheless, included this design in the experimental groups to investigate the effects of such discontinuities on the mechanical performance of soft–hard interfaces. We included a control group without gradient transitions (${W}_{\rm {{G}}}=0\,{{{{{\rm{mm}}}}}}$) (Supplementary Fig. 1a). Furthermore, to measure the morphological features of each interface, we calculated the ${A}_{\rm {{c}}}$ across the gradient lengths (Supplementary Fig. 1b). Finally, we projected each design into the narrow-gauge region of the tensile test specimens. Three specimens from each design were 3D printed, resulting in a total of 48 specimens.

### Mechanical testing and post-processing

After manufacturing, we performed quasi-static uniaxial tensile tests with a mechanical testing bench (LLOYD instrument LR5K, load cell = 100 N) at a rate of 2 mm/min until failure. The device measured the displacements ($u$), forces ($f$), and time ($t$) at a sampling rate of 100 Hz. For all the tensile test specimens, we obtained full-field strain maps (the equivalent von Mises strains) at a frequency of 1 Hz using a 3D DIC system (Q-400, two cameras each with 12 MPixel, LIMESS GmbH, Krefeld, Germany) and its associated software (Instra 4D v4.6, Danted Dynamics A/S, Skovunde, Denmark). We, therefore, painted all the specimens white, followed by the application of a black dot speckle pattern.

To generate the stress–strain curves, we defined virtual extensometers at the center of the soft section of every specimen using the DIC software and extracted the vectors of true (logarithmic) strains ($\epsilon$). We then post-processed these vectors and their respective forces ($f$) in MATLAB R2018b (Mathworks, USA) to generate their true stress vectors ($\sigma=(f/{A}_{{\rm {o}}})\exp \left(\epsilon \right)$, ${A}_{\rm {{o}}}=32.512\,{{{{{{\rm{mm}}}}}}}^{2}$). From the resulting curves, we calculated the elastic modulus, *E*, as the slope of the linear region of the stress–strain curves measured between 0 and 20% strain, the ultimate tensile strength$,\,{\sigma }_{\max }$, as the maximum recorded stress, and the strain energy density, ${U}_{{{{{{\rm{d}}}}}}}$, as the area under the stress–strain curve, also known as toughness.

### Finite element analysis of the interface designs

We created quasi-static finite element method (FEM) models of our designs using a commercially available software suite (nonlinear solver, Abaqus Standard v.6.13, Dassault Systèmes Simulia, France). Since the designs were symmetric across their length, we considered one longitudinal half of each design (Supplementary Fig. 3a). Additionally, we only included one cross-sectional unit cell of every design, yielding a perpendicular area of 24 × 24 elements. This simplification was possible because a preliminary study comparing the results of the single unit cell models with those obtained using models incorporating a full quadrant indicated that while the absolute values may be somewhat different between both types of models, the primary observations and trends remain unchanged regardless of the selected model type (Supplementary Note 2 and Supplementary Fig. 5). Given the fact that single unit cell models are much more computationally efficient, we used them for the remainder of the study to compare a large number of design alternatives. These simplifications led to discretized models with 110,592 hexagonal hybrid elements (C3D8H, enhanced hourglass control), where each voxel was represented as a single element. The hard phase was modeled as a linear elastic material with an elastic modulus $E$ of 2651 MPa and a Poisson’s ratio, $\nu$, of 0.4, while the soft phase was modeled as an Ogden hyperelastic material with the following material parameters: $N=1,\,{\mu }_{1}=0.266\,{{{{{\rm{MPa}}}}}},\,{\alpha }_{1}=3.006,\,{D}_{1}=0.113$. Finally, we applied a surface traction of ${\sigma }_{{\rm {{FEM}}}}=0.186\,{{{{{\rm{MPa}}}}}}$ to the hard end-surface and symmetric boundary conditions (i.e., ${U}_{x}={R}_{y}={R}_{z}=0$) to the soft end-surface of the mesh. The computationally predicted distributions of the von Mises strains, obtained from each of the eight integration points of every element, were used for validation and analysis. To corroborate the computational results, we compared the strain fields pertaining to the first transverse layer in the FEM models with those measured using DIC and obtained the ordinary coefficients of determination (*R*^2^) for each design. The agreement between the surface strains obtained computationally and experimentally (Supplementary Fig. 4) confirmed the validity of the computational models and motivated the use of the full 3D strain distributions resulting from FEM simulations for a more complete analysis of each soft-hard interface.

### Determination of maximum equivalent strains and strain concentration parameters

We used the true von Mises strains obtained from the DIC measurements and FEM simulations to study how the strains concentrated at the interfaces. To this end, we extracted the curves of the maximum equivalent strains ${\epsilon }_{{\rm {{eq},m}}}(x)$ from every cross-section layer across the length of the specimens. Additionally, we extracted the maximum strain value from these plots (${\epsilon }_{\max }$) and divided it over the average equivalent strain at the center of the specimen (${\epsilon }_{{{{{{\rm{N}}}}}}}$) to define the single-valued strain concentration parameter (${\epsilon }_{\rm {{c}}}={\epsilon }_{\max }/{\epsilon }_{{{{{{\rm{N}}}}}}}$) (Fig. 2a), which we obtained from the DIC measurements and FEM estimations. We used the strain values corresponding to a stress magnitude of $\sigma=0.186\,{{{{{\rm{MPa}}}}}}$. To validate our simulations, we compared the DIC-measured ${\epsilon }_{{\rm {{eq},m}}}(x)$ and ${\epsilon }_{\rm {{c}}}$ values with those calculated within the first (i.e., surface) layer of the FEM models. The good agreement between the computational and experimental results at the surface of the specimens encouraged us to use ${\epsilon }_{{\rm {{eq},m}}}(x)$ and ${\epsilon }_{\rm {{c}}}$ within the entire 3D geometry of the computational models for further analyses. Overall, these strain concentration parameters and the FEM-predicted strain distributions were used to study how the local geometry affected the performance of the functional gradients within the elastic loading regime.

### Determination of the elastic modulus functions

We used the results from our computational models to study the elastic behavior exhibited by various designs. Towards this goal, we idealized the interfaces as linear systems of springs, where the elastic modulus function ($E(x)={\sigma }_{{{{{{\rm{FEM}}}}}}}/{\epsilon }_{{{{{{\rm{avg}}}}}}}(x)$) is equivalent to the applied surface traction on the system, ${\sigma }_{{{{{{\rm{FEM}}}}}}}$, over the average strain, ${\epsilon }_{{{{{{\rm{avg}}}}}}}(x)$, of every transversal layer over each interface point (Supplementary Fig. 3b). We used these estimated functions to study how different factors, including the elastic modulus and overall rigidity, affect the performance of the interfaces.

### Quad-lap shear designs, testing, post-processing, and finite element analysis

We extended our analyses to study the shear response of the designed architectures using quad-lap shear test specimens. We chose the designs that performed the best in the tensile tests (i.e., GY, CO, and PA, ${W}_{\rm {{G}}}=4.572\,{{{{{\rm{mm}}}}}}$) and compared them to the control design (i.e., without a gradient). Developing a geometry that enables the testing of functional gradients under shear loading was required because, to the best of our knowledge, no such standards currently exist. To obtain the appropriate dimensions of the specimens, we generated multiple geometries with the FEM software and simulated them. We used the dimensions of the specimen that yielded the elastic properties of the soft material (${E}_{{{{{{\rm{soft}}}}}}}=1\,{{{{{\rm{MPa}}}}}},\,{\nu }_{{\rm {{soft}}}}=0.49$) using only the forces and displacements of the virtual crosshead. After assigning the designs to the gradient regions, we printed these specimens (three per experimental group) and tested them under the same conditions as described above for the tensile tests. We calculated the true shear stresses ($\tau=\frac{f}{2{tW}},\,t=3\,{{{{{\rm{mm}}}}}},W=33.528\,{{{{{\rm{mm}}}}}}$) and strains ($\gamma=\frac{d}{2{H}_{\rm {{S}}}},\,{H}_{{\rm {S}}}=3.048\,{{{{{\rm{mm}}}}}}$) with the force and displacement vectors extracted from the mechanical testing machine. We then calculated the shear modulus ($G$, the initial slope of the stress–strain curves measured between 5% and 35% shear strain), maximum shear strength, ${\tau }_{\max }$, and shear strain energy density, ${U}_{\rm {{d}}}$, as the area under the shear stress-strain curve of each test. Furthermore, we performed FEM simulations of each design under similar conditions as described above for the modeling of quasi-static tensile tests. In this case, the unit cell used in the gradient region had a perpendicular area of 36 × 36 elements and a length of 144 elements (with no symmetry assumed), resulting in 186,624 hexagonal hybrid elements (C3D8H) per simulation. We assigned linear elastic (i.e., $E=2651\,{{{{{\rm{MPa}}}}}},\,\nu=0.4$) and hyperelastic (i.e., Ogden, $N=1,\,{\mu }_{1}=0.266\,{{{{{\rm{Mpa}}}}}},\,{\alpha }_{1}=3.006,\,{D}_{1}=0.113$) properties to the hard and soft elements, respectively. Furthermore, we applied a shear deformation to one of the end-surfaces of the mesh (i.e., ${U}_{x}=0.4257\,{{{{{\rm{mm}}}}}},{R}_{x}={R}_{y}={R}_{z}=0$) while constraining all the displacements of the other. The results of the computational analysis were then used to calculate the strain concentration parameters of each design and for comparison with the experiments.

### Design of a hybrid design based on our final findings

We further extended our analyses to exploit the full bioinspired potential of soft–hard interfaces. To do so, we combined multi-scale hierarchical organization, crack deflection mechanisms, functional gradients, and smooth contact areas to generate a hybrid design. We selected and combined the best-performing designs from the initial analysis, defining a PA structure in which the particles were randomly scattered within a GY architecture, resulting in the GP group. To create a less stiff elastic modulus function, we decreased the densities of the hard phase, $\rho$, by 50%. We then 3D printed, tested, and computationally analyzed these designs under the same conditions as described above for the quasi-static tensile tests.

### Supplementary information

Supplementary Information

Description of Additional Supplementary Files

Supplementary Movie 1

Supplementary Movie 2

Supplementary Movie 3

Supplementary Movie 4

Supplementary Movie 5

Supplementary Movie 6

Supplementary Movie 7

Supplementary Movie 8

Supplementary Movie 9

Supplementary Movie 10

Supplementary Movie 11

Supplementary Movie 12

Supplementary Movie 13

Supplementary Movie 14

Supplementary Movie 15

Supplementary Movie 16

Supplementary Movie 17

Supplementary Movie 18

## Data Availability

The raw and processed data used in this study will be made available upon request from the lead contact.

## Code availability

The codes used to generate the composite designs and computational simulations will be made available upon request from the lead contact.
