# Supplementary material for: Bioinspired rational design of bi-material 3D printed soft-hard interfaces
Source: Nat Commun. 2023 Dec 12;14:7919. doi: 10.1038/s41467-023-43422-9 (PMC10716482; doi:10.1038/s41467-023-43422-9)
Supplement: Supplementary file 1 — Supplementary Information [file 41467_2023_43422_MOESM1_ESM.pdf]

Supplementary document to

Bioinspired rational design of bi-material 3D printed soft-hard interfaces

M. C. Saldívar[a†], E. Tay[a†], A. Isaakidou[a], V. Moosabeiki[a], L. E. Fratila-Apachitei[a], E. L. Doubrovski[b], M. J. Mirzaali [a*], A. A. Zadpoor[a]

[a] *Department of Biomechanical Engineering, Faculty of Mechanical, Maritime, and Materials Engineering, Delft University of Technology (TU Delft), Mekelweg 2, 2628 CD, Delft, The Netherlands*

[b] *Faculty of Industrial Design Engineering (IDE), Delft University of Technology (TU Delft), Landbergstraat, 15, 2628 CE, Delft, The Netherlands*

[*] Corresponding author. e-mail: m.j.mirzaali@tudelft.nl.

[†] Both authors contributed equally to this study.

Supplementary Note 1

To generate the geometries of the triply periodic minimal surfaces (TPMS) designs considered here, we used the isosurface equations (U) of Gyroids, Diamonds, and Octo in their sheet form [1]. These equations across the cartesian system (x, y, z) are as follows:

$$\text{Gyroid} \quad U_{\text{GY}} = \cos(x)\sin(y) + \cos(y)\sin(z) + coz(z)\sin(x) = 0 \qquad \ldots(1)$$

$$\text{Diamond} \quad U_{DI} = \sin(x)\sin(y)\sin(z) + \sin(x)\cos(y)\cos(z) + \cos(x)\sin(y)\cos(z) + \cos(x)\cos(y)\cos(z) = 0 \qquad \ldots(2)$$

$$\text{Octo} \quad U_{\text{OC}} = 4(\cos(x)\cos(y) + \cos(y)\cos(z) + \cos(z)\cos(x)) - 3(\cos(x) + \cos(y) + \cos(z)) = 0 \qquad \ldots(3)$$

For each of these designs, we defined the isosurfaces with the help of discrete 3D mesh vectors in MATLAB R2018b (Mathworks, USA), where each of the individual elements had the size of a voxel and the total edge of each cubic unit-cell was 2.032 mm (or 24 voxels). We then discretized the equations based on the required volume fraction of the hard material, $\rho(x)$, across the horizontal direction (x), yielding the desired 3D TPMS interface designs.

At the same time, we used the simple parametric equations of a helix for the design of the collagen-inspired specimens[2]. We defined these equations in the cartesian system as:

$$x = ct; \quad y = r\sin(t+\phi); \quad z = r\cos(t+\phi) \qquad \ldots(4)$$

where $t \in [0,2\pi)$ defines the unit-cell discretization, $c = \frac{2.032}{2\pi}$ mm is a constant that describes the separation of the helix, $r = 0.508$ mm is the radius of the helix, and $\phi = \left[0, \frac{1}{3}, \frac{2}{3}\right]\pi$ is the phase angle that defines the rotations of each of the three helices in the design. After repeating each of these cubic 2.032 mm edged unit-cells across the total dimensions of the interface, we used the $\rho(x)$ function to define the thickness of the helical beams, thereby achieving the proper material discretization.

Supplementary Note 2

We processed an additional set of simulations to study the effects of the number of modeled unit cells on the simulation results. In particular, we tried to establish whether the models in which only a single unit cell was included could capture the behavior of the actual experimental tensile tests. To this end, we prepared simulations of 3 different designs. These were the non-graded control (W_G = 0 mm), the long OC (W_G = 12 mm), and the long PA (W_G = 12 mm) (Supplementary Figure 5). We selected these designs because they are quadrant-symmetric, allowing us to simulate only 2 unit cells of the interface (*i.e.*, 1 along their thickness and 2 across their width). These simulations were prepared under the same conditions as described in the main article. However, symmetric boundary conditions were additionally applied on the $y = 0$ (*i.e.*, $U_y = 0, R_x = 0, R_z = 0$) and $z = 0$ planes (*i.e.*, $U_z = 0, R_y = 0, R_z = 0$).

The estimations made by the quadrant symmetric (Quad) model of the control group were remarkably similar to those of the single unit cell (UC) models and DIC-measured strain distributions (Supplementary Figure 5a). In both types of simulations, the deformations were highly concentrated at the interface, with the Quad and UC estimations showing ϵ_c values of 2.28 and 1.7, respectively. Although the absolute strain values differed between both types of models, they occurred at the corners of the specimens regardless of the model type. The corners of the specimens are the locations where high values of shear deformation tended to be present. These peak strain values also appeared in the DIC measurements, where, as expected, the ϵ_c value was lower than those predicted computationally. This is due to the averaging effects caused by the lower resolution of the DIC measurements as compared to the computational models and the potential photopolymer mixing prior to curing. Finally, both UC and Quad simulations showed diminished values of the von Mises strains at the center of the cross-

sections of the specimens, which is expected because, in this region, only volumetric strains occur.

In the case of the long OC geometries, the strain patterns of the first layer of the Quad and UC simulations were almost identical to those of the DIC measurements (Supplementary Figure 5b). Similarly, the patterns of the $\epsilon_{eq,m}(x)$ distribution within the first layer of the specimens presented the same transitions as observed in the experimental results, indicating that both types of models capture the essential features of the experimental observations. Consistent patterns of strain were also present within the layers with the maximum strain value, regardless of the type of the FEM model. The FEM-predicted ϵ_c values were 6.36 and 4.53 for the Quad and UC simulations, respectively. Although the magnitude of these strain peaks differed, the shapes of the maximum $\epsilon_{eq,m}(x)$ plots followed the same trends, with the strain peaks occurring in the regions with a hard discontinuous transition from the hard to the soft material. Similarly, the $E(x)$ functions estimated by both UC and Quad models were similar both in values and trends, indicating that the number of the modeled unit cells does not alter the essence of the predicted mechanical behavior.

Considering the long PA simulations, both Quad and UC models estimated somewhat higher peak strain values within the top layer of the interface than those measured through DIC (Supplementary Figure 5c). As discussed in the main text of the article, the absence of these peaks in the DIC images was likely due to its lower resolution and the potential material mixing between single droplets of the photopolymers. Despite these differences, the $\epsilon_{eq,m}(x)$ values and their localizations were remarkably similar in both the Quad and UC models. Similar to both other designs, the strain values predicted by the Quad models were higher than those resulting from the UC simulations ($\text{FEM (max)}\ \epsilon_c$ = 1.84 and 1.79 for the Quad and UC models, respectively). In both cases, such strains were localized within the middle region of the interfaces and along the edges of the specimens. As described in the main text, the strain

peaks observed within the regions made from mostly hard material are of relatively minor importance for the PA designs because any cracks initiated in these regions are likely to be arrested. Moreover, the plots of $\epsilon_{eq,m}(x)$ were remarkably similar in the regions closest to the soft material, indicating that the maximum strains estimated by the UC model are representative of the full specimens at regions where failure is most likely to occur. As in the case of the long OC specimens, little to no differences were present between the trends seen in the plots corresponding to the UC and Quad $E(x)$ models, further corroborating the claim that UC models can successfully capture the main mechanisms behind the observed differential mechanical behaviors of the various considered groups.

Overall, the main differences observed between the UC and Quad models were the FEM (max) ϵ_c values which were somewhat higher for the quadrant symmetric simulations. The localization of these peaks was, however, highly consistent between both types of models. Moreover, the differences in the peak strain values tended to disappear in the regions closest to the soft material, which are the most critical places (because failure due to a sub-optimal interface is likely to occur within those regions). Finally, the similarity between the elastic modulus functions predicted by both types of models allowed us to conclude that UC FEM models are representative of the full-size models, allowing for the application of such computationally efficient simulations for the analysis of the remaining designs.

Supplementary Note 3

An additional set of measurements were performed to compare the three-dimensional behavior of different interface designs under loading and to corroborate the performance of our computational models. For these experiments, we used TESCAN CorTOM micro-CT scanner (TESCAN, Brno, Czech Republic) for micro-CT imaging of a non-graded (Ctrl), a PA (W_G = *12* mm), and an OC (W_G = 12 mm) design. To enable in situ assessment of the deformation behavior of the specimens, we scanned the specimens under two loading conditions: without

load and under a 6 N load (Supplementary Figure 6a). A load of 6 N was selected because this was the load used for the Quad FEM simulations presented in Supplementary Note 2. The specimens were scanned over a 360˚ rotation with a 3D pixel size of 31 μm and an angular rotation step of 0.25˚. The CT images were acquired at 120 kV and 260 μA with a total duration of ~5 min per imaging cycle. The image analysis of the specimens was performed using the software Fiji (v1.53). Since no internal material pattern was present within the 3D images or any difference was present between the greyscale values of the hard and soft material regions, no digital volume correlation (DVC) analysis could be performed. However, we measured the cross-sectional areas along the length of the specimens and compared with their computational equivalents as per the FEM simulations reported in Supplementary Note 2 (Supplementary Figure 5).

When deformed, the Ctrl design presented a region with sharp deformations at the location of the soft-hard transition (Supplementary Figure 6b). Moreover, its surface area pattern under loading showed an abrupt transition of magnitude at the same region, confirming the presence of high strain concentrations at the edges of the interface. Contrary to these results, the PA and OC designs did not show the aforementioned high deformations at the corners of the interface, and their surface area plots showed a smooth transition at the interface edges. These measured surface area curves were highly similar to those estimated with the FEM simulations with smooth transitions present for the PA and OC designs and the sharp transition characterizing the Ctrl one. Furthermore, when loading the OC design, two gaps were detected in the middle of the interface when loading the specimen (Figures S4d). The locations of these gaps corresponded to the regions where the highest strain concentrations of this study were found, which was at the hard material discontinuity of the long OC interface. Overall, these results confirm that our FEM models and the study of strain concentrations at soft material regions are capable of capturing the internal 3D behavior of the presented 3D soft-hard interface designs.

Moreover, these results call for future studies with in-situ micro-CT scanning under continuous loading, where the 3D-printing equipment is modified to include micro- or nanoparticles that can be visualized with a micro-CT scanner, allowing for the recognition of the internal structure of the specimen and performing DVC.

Supplementary Note 4

We performed additional simulations to determine the failure modes that can occur within various soft-hard interface designs. These simulations were performed for the control (*i.e.*, non-graded), short (*i.e.*, W_G = 4 mm) GY, long (*i.e.*, W_G = 12 mm) CO, PA, and GP designs with the same meshes as the ones used for the initial hyperelastic simulations. In this case, however, we introduced plasticity and ductile damage with element deletion into the material models used for the soft material, allowing us to study the failure route of each design. These failure considerations were chosen since they yield an efficient and simple method to analyze the failure mode of the interfaces at multiple voxel locations without a need for predefining a single propagating crack or for including cohesive zone elements between every hard and soft voxel of the interface. Due to software (*i.e.*, Abaqus) limitations, such models required changing the elastic material properties of the soft material from hyperelastic to linear elastic (*i.e.*, elastic modulus E_{soft} = 1.3 MPa and ν_{soft} = 0.48) and the type of analysis from quasi-static to dynamic explicit. The yield stress point of the soft material was set to 1.2 MPa under a von Mises yield criterion, followed by a linear strain hardening modulus of 0.58 MPa, where element deletion was set for a plastic strain of 5% (Supplementary Figure 10a). These values are based on the existing coarse-graining models for these materials [3]. Regarding the boundary conditions, we applied a displacement to the hard edge of the specimens until mesh separation while applying symmetric boundary conditions to the soft edge of the mesh.

Following the post-processing stage (Supplementary Videos 10 to 14), we found that the stress *vs*. strain curves of all the designs show similar increases in the stress followed by failure due

to element deletion (Supplementary Figure 10b). The long GP design showed the highest values of the strain energy density, while the short GY design underperformed when compared to the other designs (Supplementary Figure 10c). Regarding the plastic energy dissipation prior to failure, the non-graded design showed hardly any capacity for energy dissipation (Supplementary Figure 10d), instead featuring high strain concentrations at the edges of the interface, which caused its sudden failure (Supplementary Figure 10e). Similarly, in the short GY models, the sharp-ended features at the edges of the specimens introduced strain concentrations that caused the separation of the mesh (Supplementary Figure 10e), confirming the design guideline presented in the main text that recommends avoiding such features. It is, however, important to emphasize that the short GY designs showed the lowest strain concentration values among all the TPMS designs in the initial hyperelastic FEM simulations. Indeed, potential photopolymer blending effects within the 3D-printed specimens may have ameliorated the negative effects of these features, explaining their improved performance in the initial experiments. The long CO and GP designs showed comparatively high strains along their interfaces with regions of relatively low values of strain concentrations that resulted in multiple regions of plastic deformations, confirming how relatively compliant transitions can help in accommodating strains through plastic energy dissipation and can prevent the catastrophic failure of more periodic interfaces. Moreover, both the PA and GP designs showed multiple regions of soft material separation along their interface prior to failure, where cracks were arrested by neighboring hard material voxels. These observations confirm how particle-based designs provide the additional toughening mechanism of crack deflection. However, it is important to mention that for such a mechanism to be applicable, the distribution of the randomly positioned particles must be periodic [4], unlike in these simulations where particles were present only in a very small cross-section area. All in all, this comparative study allowed us to study how the presented design guidelines that suggest avoiding sharp-ended geometries

and including compliant transitions and crack-arresting features have the potential to positively affect the performance of soft-hard interfaces.

Supplementary Note 5

We included additional quasi-static hyperelastic computational simulations to analyze the effects that the elastic modulus of the hard material has on the strain concentrations of the interfaces. These simulations were performed for three different interface designs (*i.e.*, GY $W_G = 4$, CO $W_G = 12$, and PA $W_G = 12$) under the same conditions as presented in Section 2.4 of the main text. Four values of the elastic modulus were tested (*i.e.*, $E_{\text{Hard}} = [250, 1000, 2651, \text{and } 10000]$ MPa).

After post-processing, we found that, as the E_{Hard} values are varied, there are only limited changes in the overall ϵ_c peak values corresponding to each design (Supplementary Figure 11). For the CO design, the changes in the properties of the hard material only affected the magnitude of the ϵ_c transition function along the gradient, which increased when decreasing E_{Hard}. The general patterns of strain distribution, however, remained consistent. The location of the ϵ_c peaks remained in the concave and sharp regions of the short GY specimens, within the soft regions of each helix in the CO specimens, and in the single soft material particles located along the interface of the PA specimens. These results indicate that even though the properties of the hard material influence the interface behavior to some extent, the overall performance of the interfaces is primarily dependent on the geometrical aspects of the functional gradients.

Supplementary Note 6

Additional analyses were performed to unravel the potential effects of photopolymer blending on the performance of soft-hard interfaces. These comprised a characterization of the size of

the zone in which the different photopolymer phases were mixed as well as additional ductile fracture computational simulations that considered photopolymer blending.

The mechanical characterization of the soft-hard mixing zone was performed using atomic force microscopy (AFM, JPK Nanowizard 3, Berlin, Germany) on a non-graded specimen (Supplementary Figure 12a). We utilized a TESP-HAR probe (Bruker, Billerica, Massachusetts, USA) with a length of 125 μm, a width of 40 μm, a thickness of 4 μm, and a nominal spring constant of 42 N/m to measure the elastic modulus change at the transition between the hard and the soft 3D printed material. The calibration of the probe was performed using the thermal tuning method and resulted in a sensitivity of 26.63 nm/V and a stiffness constant of 49.953 N/m. We indented an area of 250 × 100 μm^2 with an indenter separation of 0.39 μm, yielding 250 × 100 indentation force-displacement curves. The indentation curves were acquired for a force setpoint in the range of 1 – 4 μN. For each curve, the elastic modulus was calculated using the Hertz-Sneddon model, assuming the AFM tip to be a flat cylinder with a tip radius of 10 nm (the nominal value) with the following relation:

$$F = \frac{E}{1 - \nu^2} 2R\delta \qquad \ldots(11)$$

, where E is the elastic modulus, v is the Poisson's ratio, R is the nominal tip radius, and δ is the displacement. After processing the data, a sigmoidal function was used to characterize the mixing zone (Supplementary Figure 12b), similar to what other studies have proposed [5]. This function is defined as:

$$E(x) = \frac{E_{AFM,h}}{1 + \exp\left(-\beta(x - \gamma)\right)} + E_{AFM,s} \qquad \ldots(12)$$

, where $E(x)$ is the elastic modulus along the interface, $E_{AFM,h}$ and $E_{AFM,s}$ are the hard and soft material elastic modulus values, respectively, and β and γ are constants. $E_{AFM,h}$ and $E_{AFM,s}$ were calculated from the average E value of the farthermost 10 μm from their respective sides of the interface. The values of β and γ were obtained by fitting the function using the nonlinear

least-squares method. The size of the mixing zone was calculated as the distance between the regions exhibiting elastic moduli corresponding to 0.5% and 99.5% of the elastic modulus of the hard material in the sigmoid design. The obtained size was 151.56 μm, which is in good agreement with the ~150 μm transition reported in a previous study [5]. Moreover, 90% of the material transition (*i.e.,* 5% to 95% hard material) occurs in a span of 84.37 μm, which matches the nominal dimension of each voxel (*i.e.,* 84 μm)..

Additional ductile fracture analyses were performed to unravel the potential effects of photopolymer blending on the performance of soft-hard interfaces. Toward this end, we introduced an algorithm that allowed us create an intermediate phase between the soft and hard phases throughout the entire geometry of the specimens in our computational models (Supplementary Figure 12c). For this intermediate phase, we first changed the element-to-voxel ratio from 1 to 27 (3×3×3). To consider the potential blending at the edges of each voxel [5]. We also updated the value of the ratio of the hard material ρ'_{ijk} of each element across the ijk directions (*i.e.,* initially $\rho' = 0$ for pure soft and $\rho' = 1$ for pure hard material) by averaging it with its adjacent elements. This averaging was performed using the following equation:

$$\boldsymbol{\rho'_{i,j,k}} = \frac{1}{27}\sum_{m=-1}^{1}\sum_{n=-1}^{1}\sum_{o=-1}^{1}\rho'_{i+m,j+n,k+o} \quad \ldots(13)$$

The resulting ρ' values of each element, which were in accordance with the mixing zone fit presented above (Supplementary Figure 12d), were then used as input for obtaining the mechanical properties of each element. For this purpose, coarse-graining models for large deformations similar to those existing in the literature were used as the constitutive models [3]. We applied this process for the non-graded control design and the long PA ones and compared their resulting mechanical response with those of their non-blended variants. Since increasing the element to voxel representation by 27 would make the computational models infeasible to

run, we restricted the meshes to interfaces of 12×12 voxels in the surface area and 96 voxels in length, yielding 373248 C3D8 elements per mesh. The resulting four simulations were processed under the same conditions as described in the Supplementary Note 4 of this document.

After post-processing the results (Supplementary Videos 15-18), the blended version of both the control and the PA gradients showed improvements in their ultimate stress and strain energy density (Supplementary Figure 12 e-g). For the non-graded design, the strength increased from 0.382 MPa to 0.399 MPa, while the strain energy density increased from 20.04 kJ/m^3 to 22.37 kJ/m^3. As for the PA, the strength increased from 0.41 to 0.45 MPa, whereas the strain energy density improved from 35.96 to 44.6 kJ/m^3. The relatively larger improvements observed in the particle-based design can be partially attributed to the increased contact surface area between the polymer phases. It is, however, important to acknowledge that the separation of the blend phases highly depends on the selected coarse-graining model. In this case, we assumed that the ultimate strain before the separation of the intermediate phase is given by the rule of mixtures between the hard and soft material phases. This assumption was necessary because no experimental data exist regarding the ultimate strain of the sub-voxel blended phase. That said, the overall strain distribution of the blended simulations and the failure modes of both designs were remarkably similar regardless of whether blending was implemented in the models (Figures S13h). In the case of the PA designs, multiple non-critical cracks were present prior to failure for both simulations, indicating that the crack deflection capacity of this design remains present regardless of whether blending is implemented in the models.

In short, these analyses showed that while photopolymer mixing plays a potential toughening role by reducing interfacial strain concentrations, the overall behavior and the toughening mechanisms achieved by varying the geometry of the interface remain consistent between the models incorporating such a blending effect and those neglecting it. To properly account for

the additional toughening mechanisms resulting from blending, one would need to perform additional experimental characterizations of the effects of photopolymer blending on the complex particle-based composites, building up on this and other existing analyses of soft-hard slab connections [5,6].

Supplementary Note 7

Studying fracture toughness, which indicates the amount of stress required to propagate a predefined flaw, requires testing specimens with a prescribed crack that is deformed until critical propagation, enabling the calculation of the *J*-integral, J_0, or the critical stress intensity factor, K_{IC}. However, to the best of our knowledge, and similar to what has been discussed for tensile and shear loading modes, there are no available standards for evaluating the fracture behavior of functionally graded interfaces. That is partially because most established standards have been developed for analyzing homogeneous and periodic materials (*e.g.,* ISO 12135) or adhesive-bonded surfaces (*e.g.,* ASTM D3762). Furthermore, the heterogeneous nature of FGs makes it necessary to change the location of the crack along the interface length and permute the interface geometry present immediately at the edge of the crack tip to find the region of the interface where the value of J_0 and K_I are the most critical. The combination of these two considerations presents an extensive and challenging task for evaluating the fracture phenomenon. Nevertheless, we have performed a pilot study to illustrate the differences between the mechanical behavior of our FGs under fracture loading conditions and to showcase the aforementioned effects of crack position and geometry at the end of the crack tip on J_0 and K_{IC}. This study was divided into two parts. For the first, a fracture toughness test specimen with a prescribed crack was used to determine the fracture toughness of multiple FGs in terms of J_0. For the second, we performed linear elastic XFEM simulations to showcase the estimated changes of the stress intensity factor, K_I, when varying the location of a prescribed crack along

the length of the gradient as well as permuting the interface geometry present at the end of the crack tip.

We defined a symmetrical hard-soft-hard interface geometry for the fracture toughness test specimens, with nominal dimensions of 24.4×24.4 mm^2 in length and width ($L \times W$), as well as an out-of-plane thickness of 24.4 mm (B) to promote critical crack propagation (Supplementary Figure 13a). The crack, with an initial length of a_0 = 6.1 mm, was prescribed at the soft end of the interface, which was the location where cracks propagated for most of the inefficient interfaces under uniaxial tensile deformations. The selected FG geometries were GY, PA, GP (W_G = 8.13 mm), and a non-graded control (Ctrl). After 3D-printing three specimens for each design, we tested them for mode I deformations using the same equipment and setup as the tensile tests described in Section 2.3 of the main manuscript. For post-processing, we calculated the fracture toughness of each test in terms of the J-integral, J_0, utilizing the standard for ductile homogeneous materials ISO 12135. This standard defines J_0 with the following expression:

$$J_0 = \left(\frac{F_{max}}{\sqrt{B^2 W}} g_2\right)^2 \frac{1-\nu^2}{E} + n_p \frac{U_p}{B(W-a_0)} \qquad \ldots(5)$$

, where F_{max} is the maximum recorded force of the test, E is the elastic modulus (*i.e.*, the slope of the stress-strain curve of the crosshead measured between 0 to 50% maximum stress), and ν is the Poisson's ratio of the specimen which is assumed to be 0.45, for simplicity. g_2 and n_p are geometrical factors defined by the following expressions:

$$g_2 = \left(2+\frac{a_0}{W}\right)\left(0.886 + 4.64\left(\frac{a_0}{W}\right) - 13.32\left(\frac{a_0}{W}\right)^2 + 14.72\left(\frac{a_0}{W}\right)^3 - 5.6\left(\frac{a_0}{W}\right)^4\right)\left(1-\frac{a_0}{W}\right)^{-1.5} \qquad \ldots(6)$$

$$n_p = 2 + 0.522\left(1-\frac{a_0}{W}\right) \qquad \ldots(7)$$

The remaining term, U_p, represents the plastic strain energy of the system and is defined in terms of the total strain energy, U, and the elastic strain energy, U_e, with the following expressions:

$$U_p = U - U_e \quad \ldots(8)$$

$$U_e = \frac{F_{max}^{\ 2}}{2K} \quad \ldots(9)$$

$$U = \int dFdCMOD \quad \ldots(10)$$

, where *CMOD* is the crack mouth opening displacement of the specimen, measured with digital extensometers within the DIC software, K is the slope of the *F-CMOD* curve measured between 0% and 50% of F_{max}, and *U* was measured as the area under the *F-CMOD* curve.

After post-processing the data, the results showed a distinct behavior between the non-graded specimens and the FG designs (Supplementary Figure 13b). The non-graded design showed the lowest mean J_0 values of the study (*i.e.,* mean = 64.07, SD = 7.6 KJ/m^2). These designs were followed by the GP (*i.e.,* mean = 70.22, SD = 5.9 KJ/m^2), the GY (*i.e.,* mean = 73.79, SD 3.9 KJ/m^2), and the PA graded design (*i.e.,* mean = 78.91, SD 7.5 KJ/m^2), which showed the highest performance. These indicate that introducing a graded interface improved the performance of the specimens by 9.6-23.1%. However, it is essential to address that, when inspecting the equivalent von Mises strain fields of the interfaces (Supplementary Figure 13c), only the control group specimens presented a secondary crack that initiated at the interface edge that was not included in the original design. This observation corroborates the observation that the unwanted strain concentrations at the edges of the soft-hard connections play a critical role in the performance of soft-hard connections. Overall, this analysis contributes to the definition a suitable geometry for studying the fracture behavior of soft-hard interfaces and confirms the improved behavior of our transition geometries as compared to their gradient-less

equivalents. However, future studies on the configuration of the fracture test specimen require testing configurations that guarantee no additional cracks are propagated during the analysis. Since it is important to prescribe the crack at a position within the interface where the outcome performance is expected to be minimal, performing a permutation analysis on the crack position through computational techniques may be necessary. We performed a pilot analysis with such a method using linear elastic XFEM simulations. These XFEM analyses comprised two different sets of tests. For the first one, we varied the position of the prescribed crack along the interface and estimated the corresponding stress intensity factor, K_I (Supplementary Figure 13d). For this purpose, the crack position was permuted between 5 voxels behind the interface (*i.e.,* the soft material side) to 6 voxels within the interface geometry (*i.e.,* toward the hard material side). For the second set of tests, the crack position was fixed at the edge of the interface geometry while the geometry of the FG was shifted between 0 and 12 voxels along the direction of the crack (Supplementary Figure 13e). Such a shift in the interface geometry allows us to evaluate how much the local material configuration ahead of the crack tip affects the K_I value. The selected interface designs for testing included OC, DI, GY, CO, PA, and GP, with the addition of a non-graded specimen as a control group. This yielded 84 XFEM simulations for the first set of tests and 91 simulations for the second. To make the computational models feasible, we discretized only one interface quadrant (*i.e.,* 144×144 voxels) and only one unit cell in thickness (*i.e.,* 24 voxels) of the fracture toughness specimen design, leading to 497664 hexahedral elements with full integration per simulation. Symmetric boundary conditions were applied at the soft material end (*i.e.,* $U_x = R_y = R_z = 0$), at the edge opposite to the interface (*i.e.,* $U_y = R_z = R_z = 0$), and at the back of the specimen (*i.e.,* $U_z = R_x = R_y = 0$), while a linear stress of 0.186 MPa was prescribed at the hard material end.

After post-processing the simulations, high variations of K_I were present when changing the crack position along the interface length (Supplementary Figure 13f). The maximum K_I value of the non-graded design was 1086 MPa.mm$^{1/2}$, estimated at one voxel within the hard material region, which was similar to the value at the edge of the interface, which was 924 MPa.mm$^{1/2}$. Contrary to this, the maximum estimated values of the FG designs varied between 472 and 646 MPa.mm$^{1/2}$, with most of these estimations being found for cracks located well within the interface geometry. Moreover, when positioned within the interface region, some of the K_I values of the FGs were as low as 3.76 MPa.mm$^{1/2}$. These differences of up to two orders of magnitude indicate a high dependence of fracture toughness value on the positioning of the crack. Similarly, shifting the FG geometry while leaving the crack stationary showed substantial variations of K_I (Supplementary Figure 13g). For this case, the behavior of all the geometries was within the range of 3 to 6 MPa.mm$^{1/2}$. However, one FG design can have a lower or higher K_I than the other designs depending on what part of its geometry is directly ahead of the crack tip. For example, a lattice shift of half a unit cell (*i.e.*, 12 voxels) of a CO design can make its K_I increase from 3.8 to 5.27 MPa.mm$^{1/2}$ and change its rank from the most efficient geometry to the fourth most efficient. Therefore, these results corroborate our hypothesis that measuring the most critical K_I of any interface design would require extensive permutation analyses to test all the possible configurations of crack positioning within a geometry, making this a highly laborious evaluation method. Consequently and as suggested in the main text, analyzing the performance of FG designs in terms of the strain concentration factor is a far more efficient methodology than the one presented in this section.

Supplementary Table 1. The quasi-static tensile test results corresponding to the various types of functional gradient (mean ± standard deviation).

Geometry	Gradient Width W_G (mm)	Elastic modulus E (MPa)	Ultimate tensile strength $\sigma_{\max}$ (MPa)	Strain energy density U_d (kJ/m^3)	DIC-measured ϵ_c (-)	FEM-estimated ϵ_c (-)
Control	0.000	0.9727±0.006	1.8336±0.084	483.112±23.90	1.5529±0.071	1.699
Gyroid (GY)	4.064	0.9313±0.022	2.6824±0.162	682.564±11.01	1.0805±0.029	2.0450
	8.128	0.9854±0.004	2.2128±0.270	531.134±53.90	0.9916±0.032	1.9967
	12.192	1.0444±0.008	2.1747±0.063	504.993±10.53	1.1638±0.048	2.4933
Diamond (DI)	4.064	1.0105±0.058	2.3957±0.203	563.165±69.80	0.9566±0.015	2.2475
	8.128	0.9963±0.037	2.399±0.431	571.999±148.84	0.9365±0.011	2.3372
	12.192	1.0497±0.040	2.3479±0.199	575.331±64.10	1.0050±0.070	2.3804
Octo (OC)	4.064	0.9959±0.028	1.7508±0.011	420.576±4.73	0.9423±0.026	1.9600
	8.128	0.9822±0.008	1.8308±0.067	430.172±4.29	1.1428±0.035	2.3228
	12.192	0.9879±0.003	1.007±0.111	216.064±25.97	1.0922±0.105	4.5312
Collagen (CO)	4.064	0.9468±0.052	2.1563±0.272	502.658±52.28	1.0295±0.035	1.6625
	8.128	0.9913±0.112	2.2817±0.164	547.302±17.12	0.9824±0.094	1.6852
	12.192	0.9755±0.051	2.6234±0.139	643.332±37.12	1.0153±0.026	1.6295
Particles (PA)	4.064	0.9730±0.117	2.1931±0.351	480.182±43.88	1.0903±0.031	1.6000
	8.128	1.0314±0.001	2.2043±0.090	500.503±14.64	1.1414±0.002	2.4305
	12.192	0.9860±0.015	2.5354±0.198	615.399±62.65	1.0682±0.044	1.7915
GY+PA (GP)	4.064	1.0154±0.053	2.3202±0.110	545.249±48.94	1.0910±0.017	2.7173
	8.128	0.9335±0.012	2.3669±0.060	630.617±51.79	1.0182±0.075	2.2441
	12.192	0.9693±0.090	2.6975±0.036	715.459±18.32	1.0421±0.025	2.0593

Supplementary Table 2. The quasi-static shear test results corresponding to the various types of functional gradients (mean ± standard deviation).

Geometry	Gradient Width W_G (mm)	Shear modulus G (MPa)	Ultimate shear strength $\tau_{\max}$ (MPa)	Strain energy density U_d (kJ/m^3)	FEM-estimated ϵ_c (-)
Control	0.000	0.3673±0.0102	3.4158±0.0960	0.8766±0.0504	2.3490
Gyroid (GY)	4.572	0.2815±0.0142	2.3868±0.0379	0.7114±0.0663	4.4659
Collagen (CO)	4.572	0.2817±0.0110	2.9269±0.0606	0.8921±0.0103	3.4884
Particles (PA)	4.572	0.3023±0.0495	3.8016±0.0415	1.1489±0.0871	2.9438

Supplementary References

1. Kapfer, S. C., Hyde, S. T., Mecke, K., Arns, C. H. & Schröder-Turk, G. E. Minimal surface scaffold designs for tissue engineering. *Biomaterials* **32**, 6875–6882 (2011).
2. Weisstein, E. W. Helix. *MathWorld--A Wolfram Web Resource* https://mathworld.wolfram.com/Helix.html.
3. Saldívar, M. C., Doubrovski, E. L., Mirzaali, M. J. & Zadpoor, A. A. Nonlinear coarse-graining models for 3D printed multi-material biomimetic composites. *Addit. Manuf.* **58**, 103062 (2022).
4. Mirzaali, M. J. *et al.* Length-scale dependency of biomimetic hard-soft composites. *Sci. Rep.* **8**, 12052 (2018).
5. Zorzetto, L. *et al.* Properties and role of interfaces in multimaterial 3D printed composites. *Sci. Reports 2020 101* **10**, 1–17 (2020).
6. De Noni, L. *et al.* Modelling the interphase of 3D printed photo-cured polymers. *Compos. Part B Eng.* **234**, 109737 (2022).

Supplementary Figures

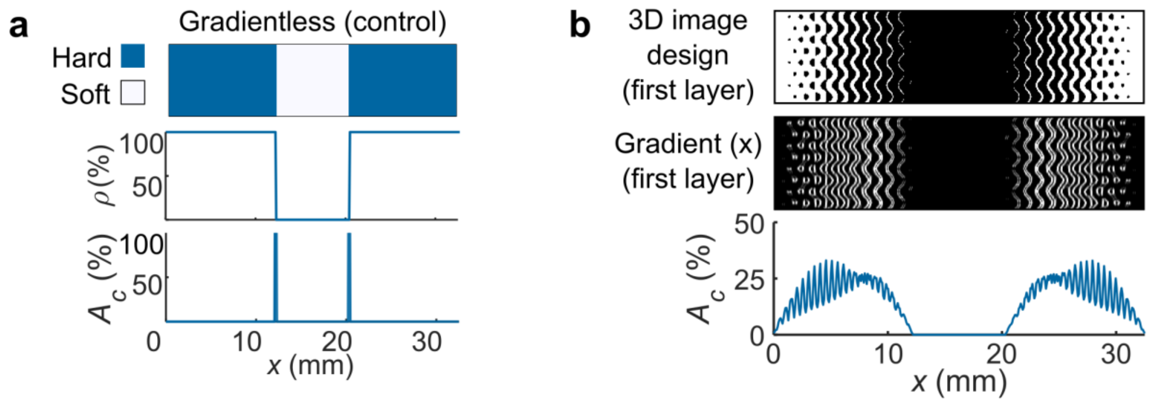

Supplementary Figure 1. The Gradientless design and contact area (A_c) calculation sketches. a) The design of the control group and its material distribution, where no gradient was considered between both phases. b) The schematic representation of the process to calculate the percentage of hard-soft normal contact area for every design. To calculate A_c, we used the 3D image gradient in the *x*-direction, which was calculated via the Sobel method and normalized the total surface area of each cross-sectional layer for every design.

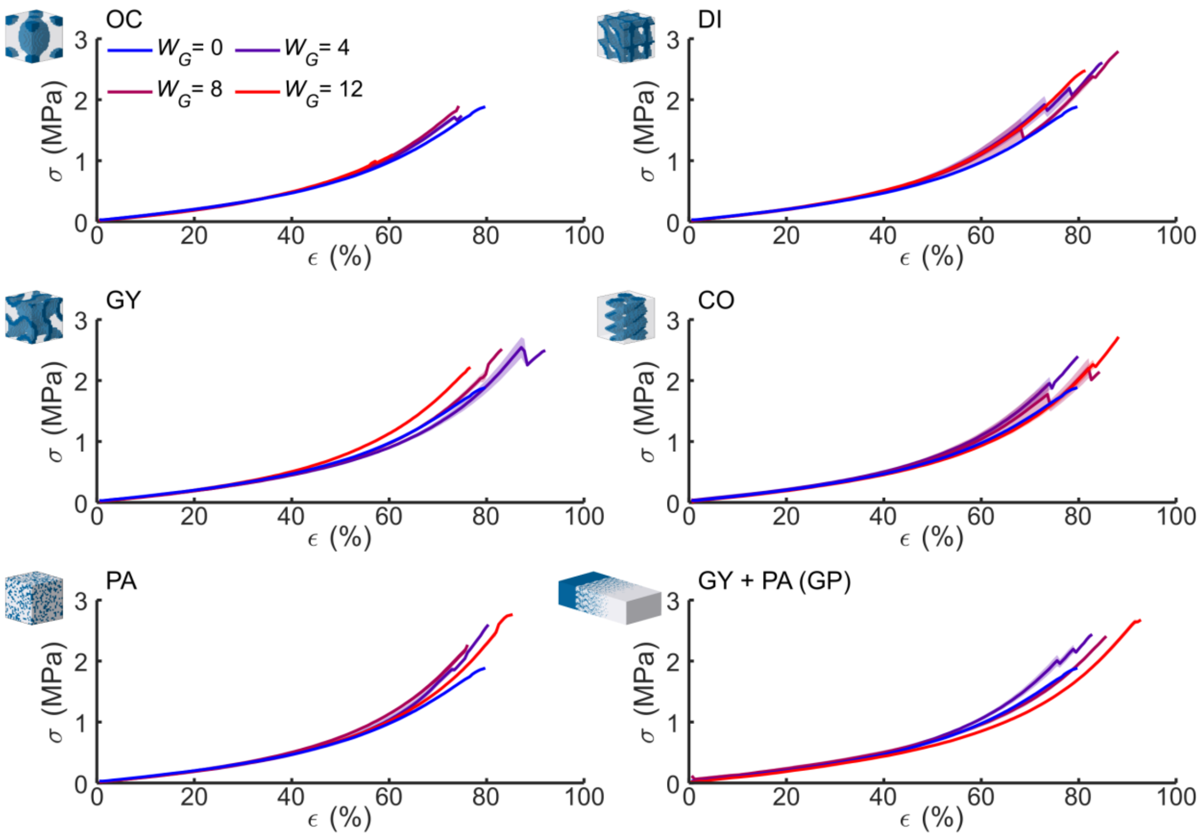

Supplementary Figure 2. The quasi-static tensile test results. The true stress *vs.* strain ($\sigma\ vs.\ \epsilon$) show the mean (and shaded $\pm$ SD) hyperelastic behavior of the tested interfaces for different gradient lengths (W_G).

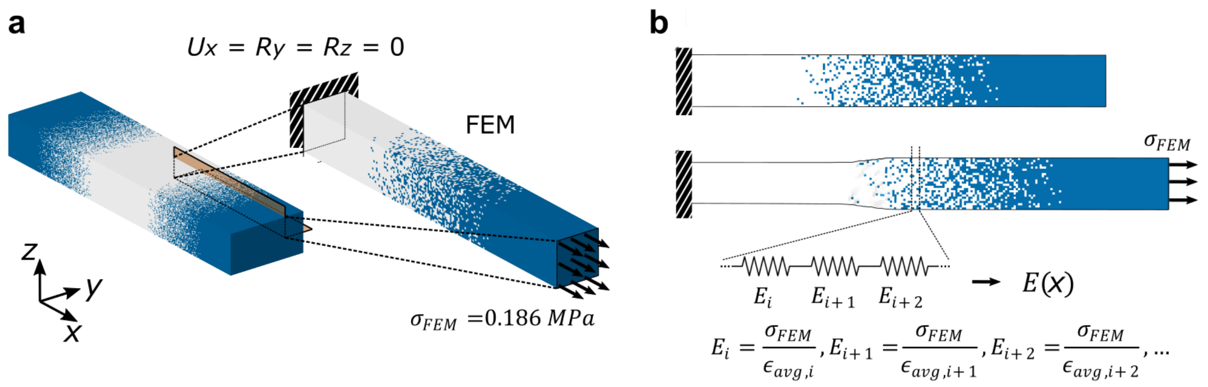

Supplementary Figure 3. The prescribed boundary conditions to the computational simulations. a) We considered one cross-sectional unit-cell (24×24 voxels) and one-half of the symmetric interface to generate the meshes of every computational simulation, where we prescribed symmetric boundary conditions ($U_x = R_y = R_z = 0$) on its soft edge and surface traction on its hard end. b) The schematic representation for calculating the elastic modulus function ($E(x)$) of every design, where we idealized each cross-sectional layer of the FGs into linear springs, from which we obtained their average deformations ($\epsilon_{avg,i}$) and elastic moduli (E_i).

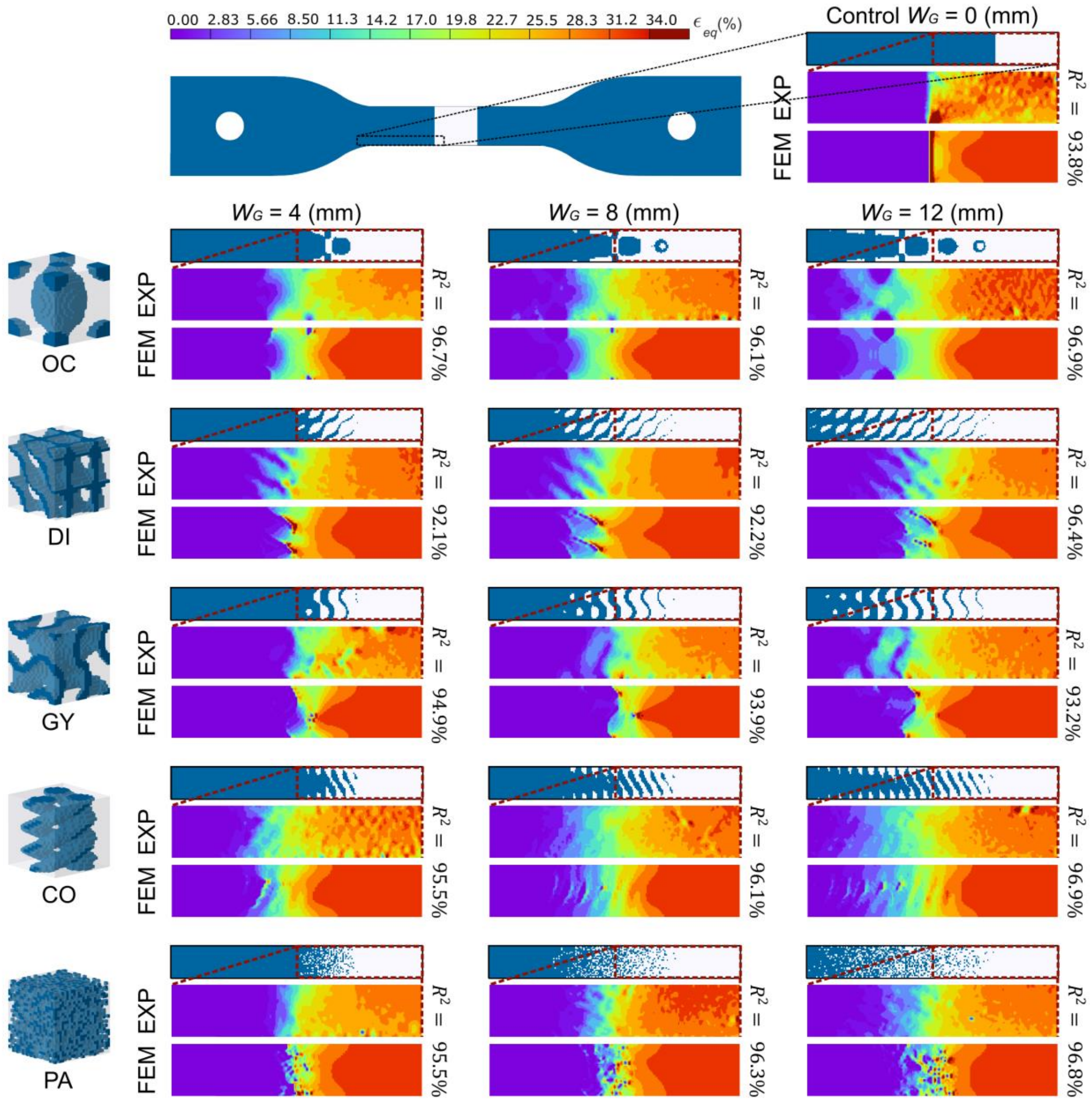

Supplementary Figure 4. A comparison between the surface strains of the experimental DIC measurements and strain of the top surface of the FEM simulations. The R^2 values represent the ordinary coefficients of determination when comparing all the strains between the images.

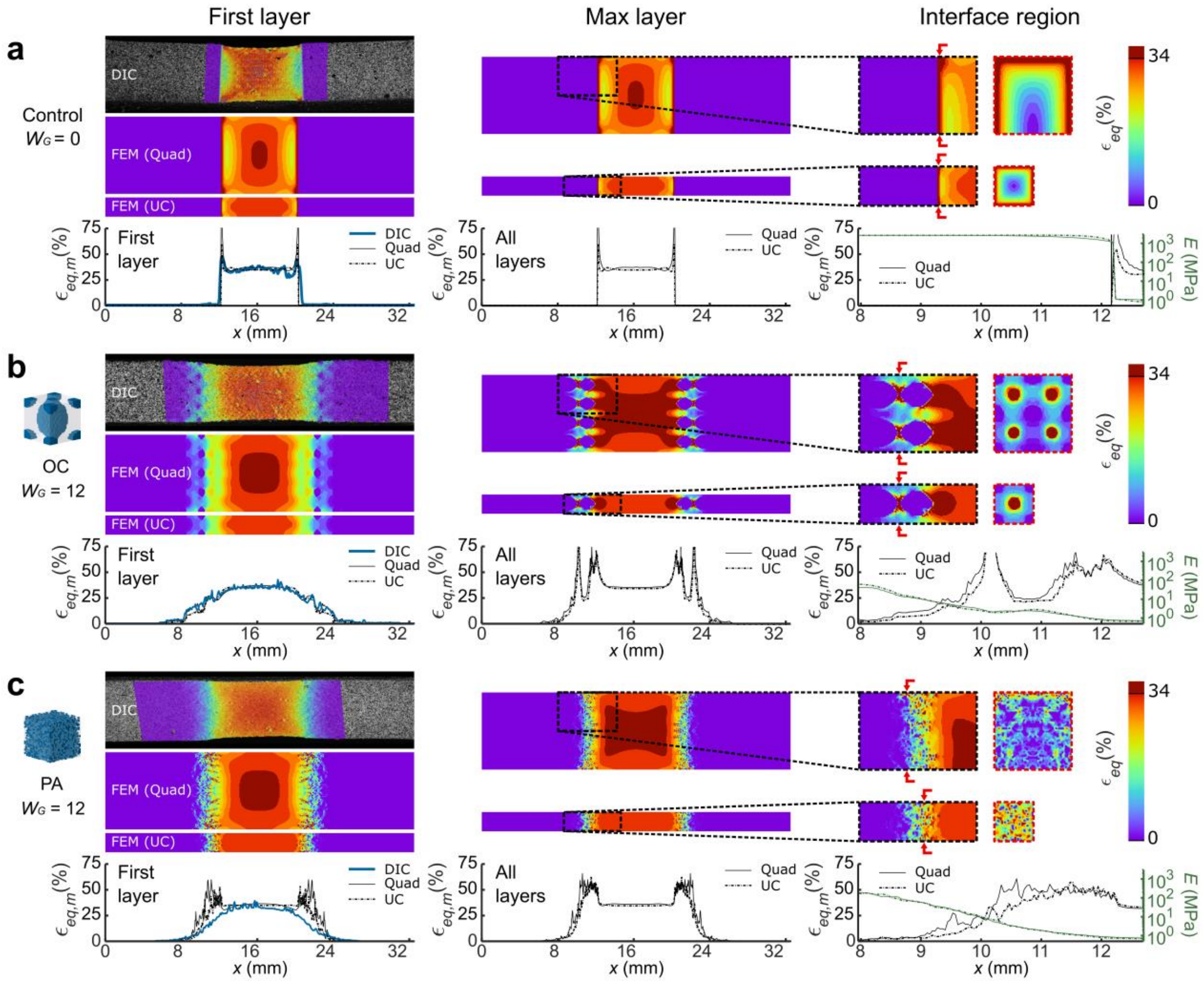

Supplementary Figure 5 A comparison between the results of the symmetric quadrant (Quad) and single unit cell (UC) FEM models. We performed detailed comparisons between the results of both types of simulations on the one hand and the DIC measurements on the other. In particular, we compared the maximum equivalent strains and the plots corresponding to the elastic modulus transitions between these simulations and the DIC-based measurements. The selected designs were the non-graded control group (a), the long OC designs (b), and the long PA (c).

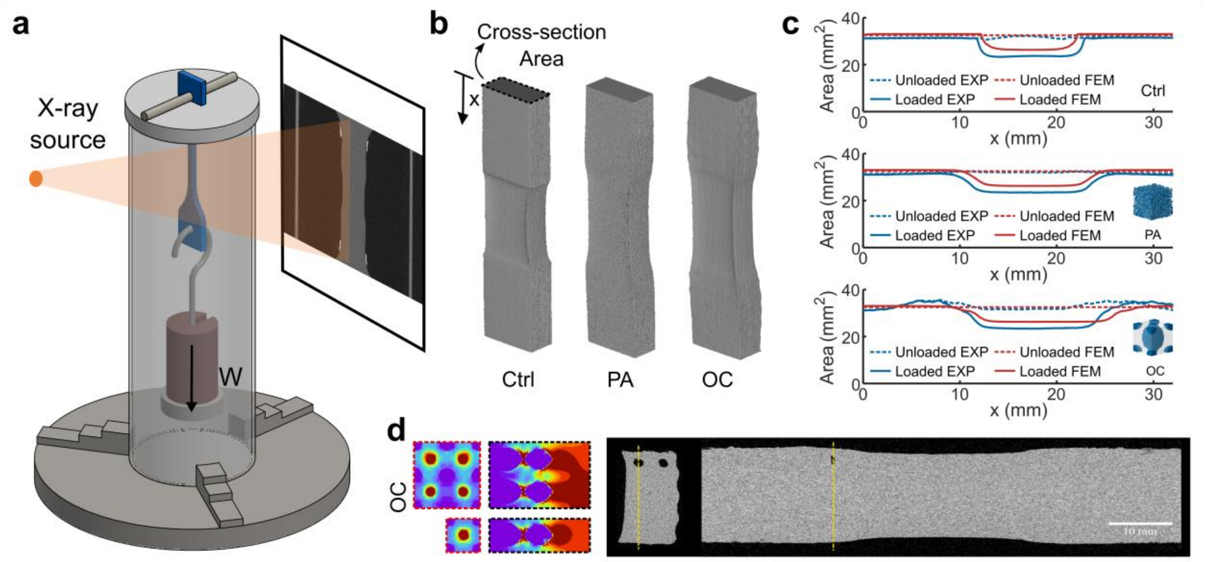

Supplementary Figure 6: The micro-CT imaging results. a) The micro-CT scan setup, where the tensile test specimens were imaged under a loaded and unloaded state to analyze their three-dimensional behavior. b) The 3D images of the non-graded (Ctrl), PA, and OC specimens when loaded with a weight *W* of 6 N, showcasing the sharp deformations at the non-graded interface and the smooth deformations of the PA and OC designs. c) The cross-section surface area plots of both specimens measured from the 3D scans and estimated with the Quad FEM simulations (Section S2). d) Cross-sectional cuts of the loaded OC specimen, showcasing the gaps initiated at the regions where the high strain concentrations of the simulations were present.

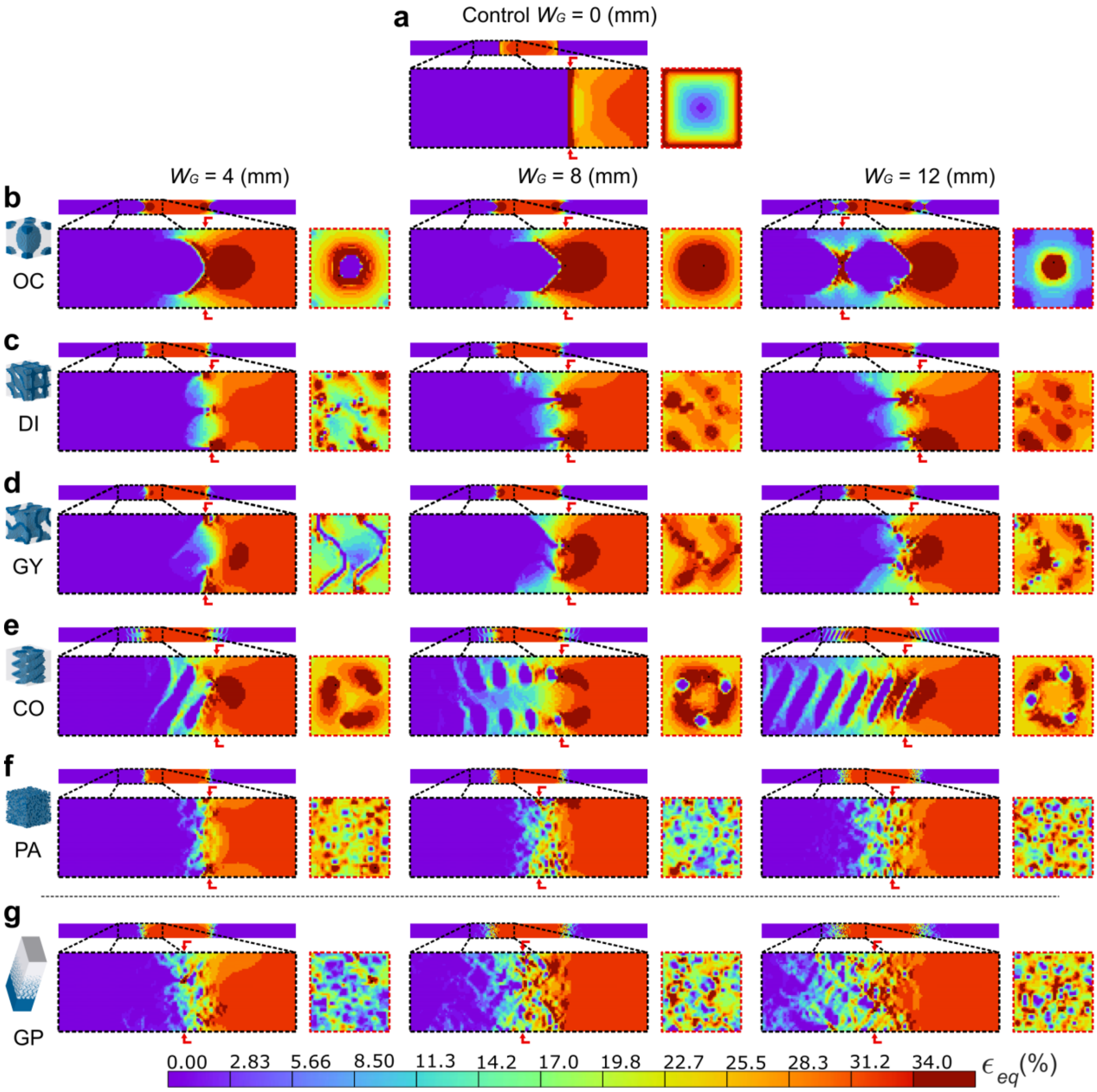

Supplementary Figure 7. The FEM-predicted distributions of the true equivalent strain of every studied interface under tensile conditions obtained from the transversal and cross-sectional layers that presented the peak strain values. The strains of every figure were obtained from the layer that presented the peak values of strain for every design. The designs were the Control (a), Octo (b), Diamond (c), Gyroid (d), collagen (e), randomly-reinforced particles (f), and Gyroid + particles interface (g).

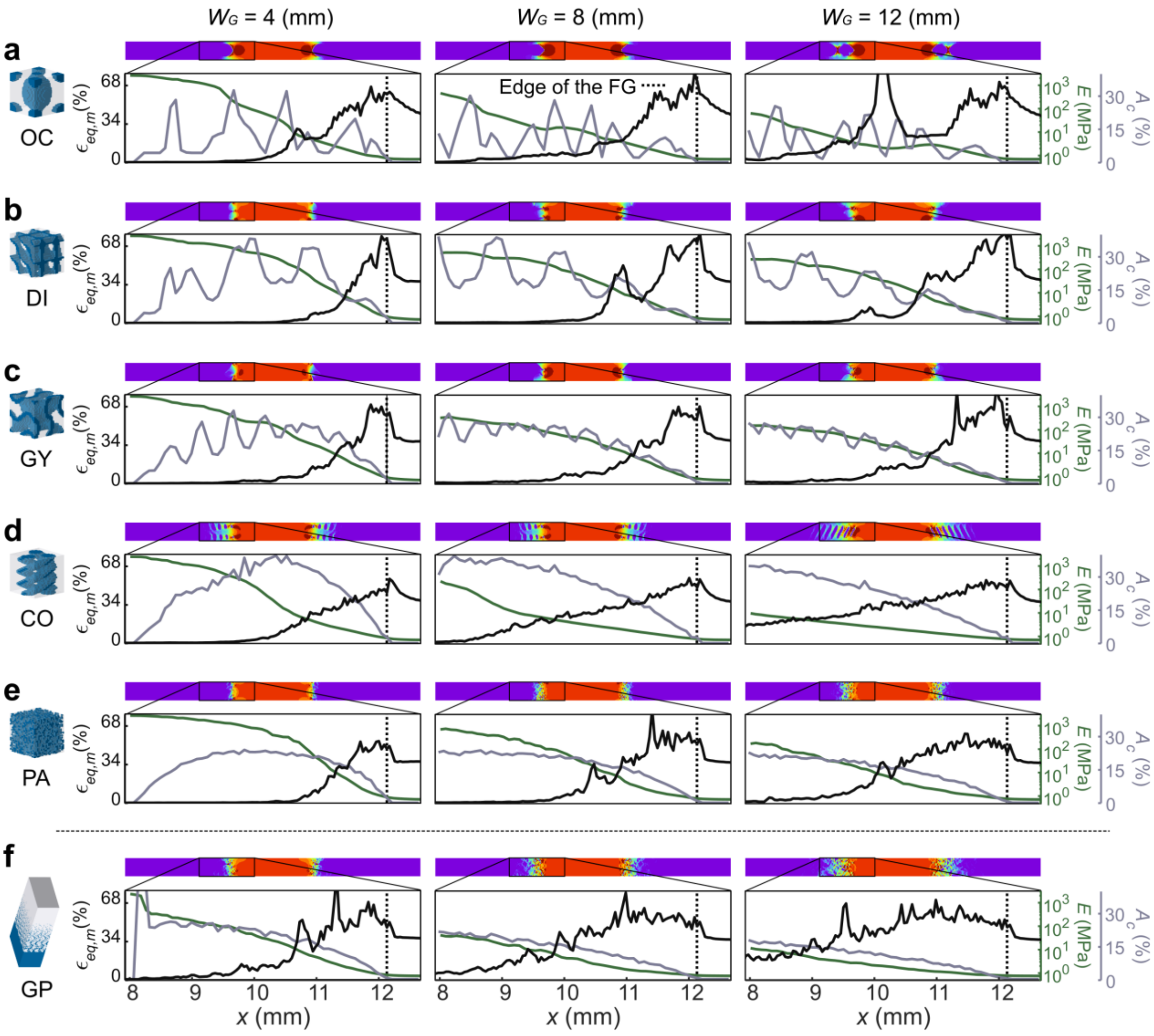

Supplementary Figure 8. Detailed plots comparing the A_c pattern and estimated elastic modulus $E(x)$ with the estimated maximum equivalent strain, $\epsilon_{eq,m}(x)$, for every interface at the final 4 mm of every studied FG. The designs were the Octo (a), Diamond (b), Gyroid (c), collagen (d), randomly-reinforced particles (e), and Gyroid + particles interface (f).

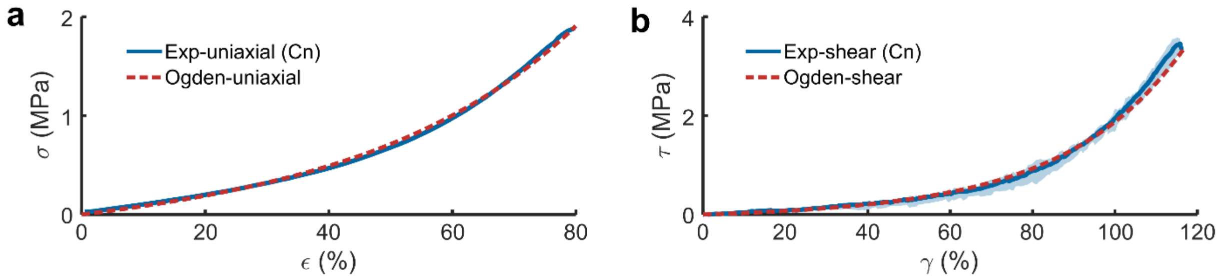

Supplementary Figure 9. The comparison between the selected Ogden model hyperelastic constitutive model vs. the mean experimental data (and shaded ± SD) of the non-graded specimens obtained under (a) uniaxial tensile and (b) simple shear loading conditions.

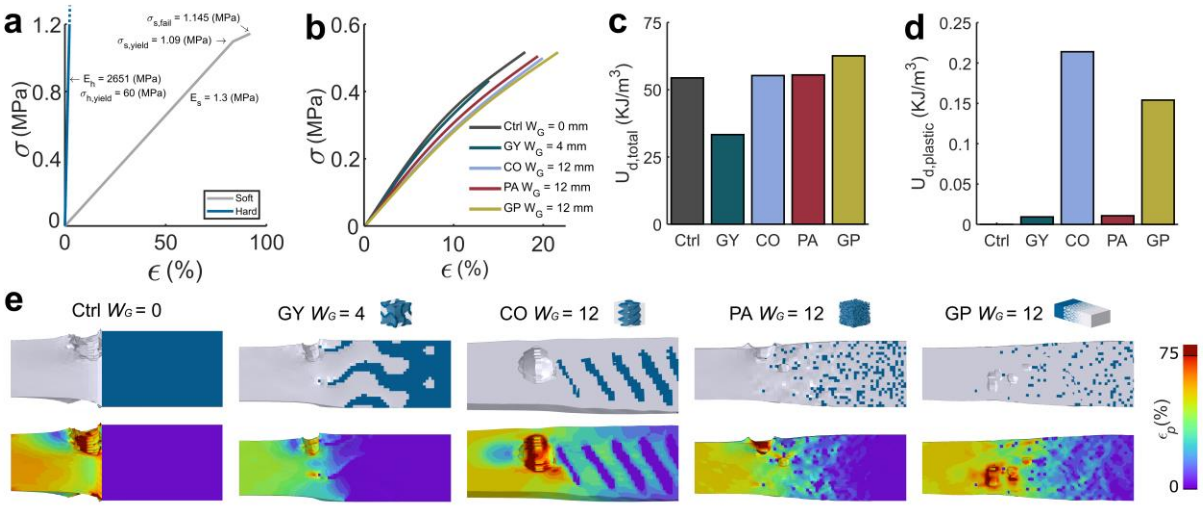

Supplementary Figure 10. The results of the ductile failure analysis applied to a selected number of designs. a) The input soft and hard material properties for this analysis. b) Stress-strain curves of all the considered designs. The values of the total (c) and plastic (d) strain energy densities. e) the distribution of the first principal strain in the simulations and the failure modes prior to critical failure.

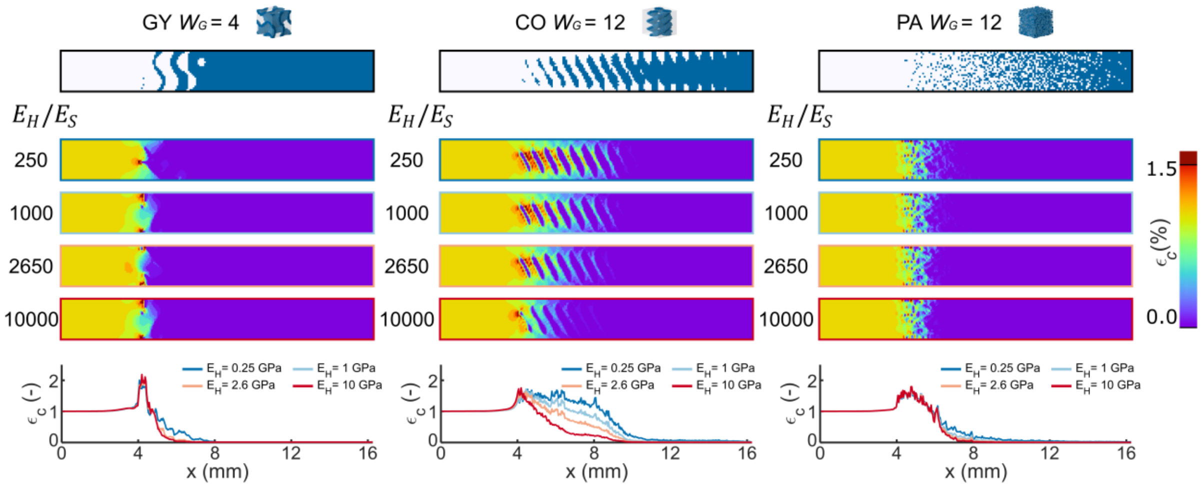

Supplementary Figure 11: The strain concentrations resulting from the different ratios of the elastic modulus of the hard material to that of the soft material. These results confirm that the maximum values of ϵ_c remain relatively similar, and the patterns of the ϵ_c transition along the interface remain consistent with the gradient geometries.

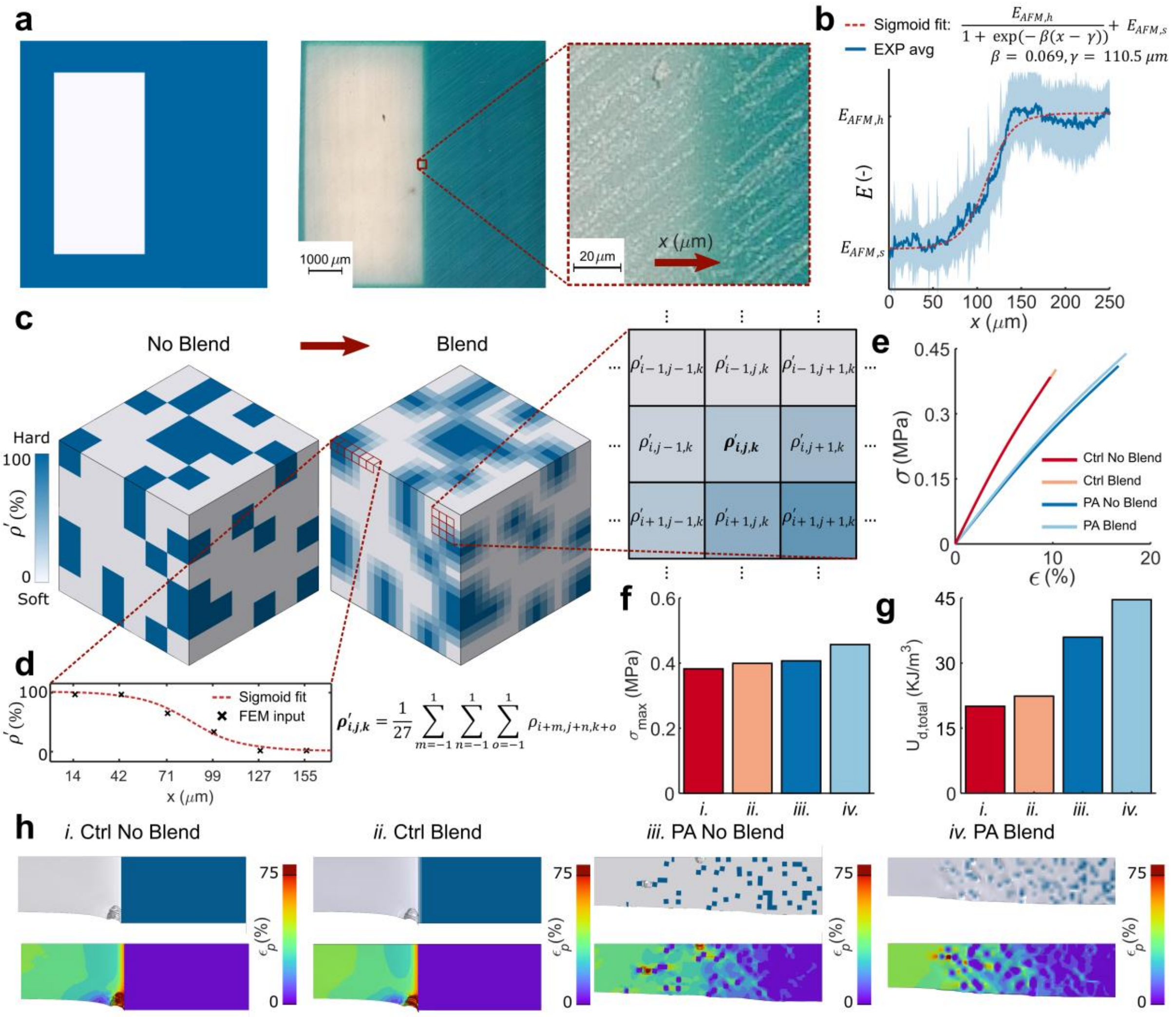

Supplementary Figure 12: The effects of photopolymer blending on the outcome of explicit ductile failure FEM simulations. a) The soft-hard non-graded interface used to characterize the size of the material mixing zone through AFM indentation. b) The resulting average values of the AFM-measured elastic modulus for the case where the transition zone was designed using a non-graded function (and shaded $\pm$ SD), which was fitted into a Sigmoid function. c) The hard material ratio (ρ') of a blended photopolymer composite is obtained by averaging the initial value of each element with those of its adjacent elements. d) The resulting ρ' values used for input in the FEM simulations closely matched those of the sigmoidal mixing zone characterization. e) the stress-strain curves, f) the ultimate stress, and g) the ultimate strain of the resulting simulations. h) the distributions of the first principal true strain of each design just before its critical failure.

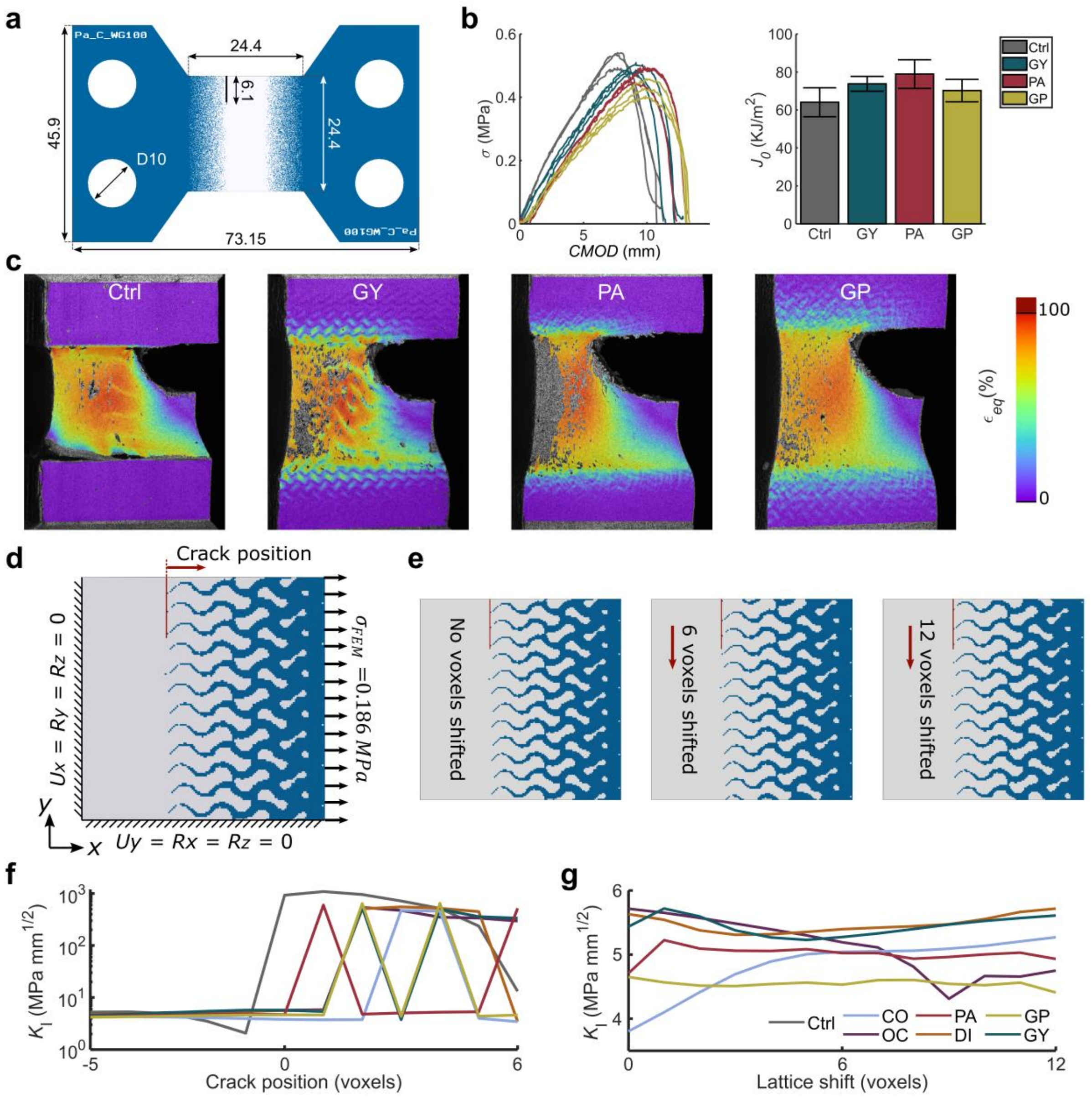

Supplementary Figure 13: The results of the fracture behavior analyses performed for multiple FG interfaces. a) The design of the fracture toughness test specimen used for the analysis, with an out-of-plane thickness of 24.4 mm. b) the stress vs. crack mouth opening displacement (*CMOD*) curves of each performed test and their respective mean ± SD *J*-integral (J_0) results. c) The equivalent von Mises strain distributions of the selected designs at the point of maximum recorded strength. d) The XFEM mesh utilized in the computational models, where the crack position was permutated along the edge of the interface. e) Representative XFEM meshes where the interface geometry was shifted ahead of the crack tip. f and g) The

resulting stress intensity factor of the interfaces when varying the position of the crack along the tip (f) and when shifting the geometry ahead of the crack tip (g).
