# Supplementary material for: Bioinspired rational design of bi-material 3D printed soft-hard interfaces
Source: Nat Commun. 2023 Dec 12;14:7919. doi: 10.1038/s41467-023-43422-9 (PMC10716482; doi:10.1038/s41467-023-43422-9)
Supplement: Supplementary file 2 — Description of Additional Supplementary Files [file 41467_2023_43422_MOESM2_ESM.pdf]

Description of additional supplementary files

Bioinspired rational design of bi-material 3D printed soft-hard interfaces

M. C. Saldívar[a†], E. Tay[a†], A. Isaakidou[a], V. Moosabeiki[a], L. E. Fratila-Apachitei[a], E. L. Doubrovski[b], M. J. Mirzaali [a*], A. A. Zadpoor[a]

[a] *Department of Biomechanical Engineering, Faculty of Mechanical, Maritime, and Materials Engineering, Delft University of Technology (TU Delft), Mekelweg 2, 2628 CD, Delft, The Netherlands*

[b] *Faculty of Industrial Design Engineering (IDE), Delft University of Technology (TU Delft), Landbergstraat, 15, 2628 CE, Delft, The Netherlands*

* Corresponding author. e-mail: m.j.mirzaali@tudelft.nl.

† Both authors contributed equally to this study.

File Name: Supplementary Movie 1

Description: The mechanical behaviour of the Octo (OC) design under tensile loading conditions.

File Name: Supplementary Movie 2

Description: The mechanical behaviour of the Diamond (DI) design under tensile loading conditions.

File Name: Supplementary Movie 3

Description: The mechanical behaviour of the Gyroid (GY) design under tensile loading conditions.

File Name: Supplementary Movie 4

Description: The mechanical behaviour of the Collagen-like helices (OC) design under tensile loading conditions.

File Name: Supplementary Movie 5

Description: The mechanical behaviour of the randomly distributed Particles (PA) design under tensile loading conditions.

File Name: Supplementary Movie 6

Description: The mechanical behaviour of the Gyroid (GY) design under shear loading conditions.

File Name: Supplementary Movie 7

Description: The mechanical behaviour of the Collagen-like helices (OC) design under shear loading conditions.

File Name: Supplementary Movie 8

Description: The mechanical behaviour of the randomly distributed Particles (PA) design under shear loading conditions.

File Name: Supplementary Movie 9

Description: The mechanical behaviour of the Gyroid + Particles (GP) design under tensile loading conditions.

File Name: Supplementary Movie 10

Description: The results of the dynamic explicit FEM simulations of the Gradientless (ctrl) design under tensile loading conditions.

File Name: Supplementary Movie 11

Description: The results of the dynamic explicit FEM simulations of the Gyroid (GY) design under tensile loading conditions.

File Name: Supplementary Movie 12

Description: The results of the dynamic explicit FEM simulations of the randomly distributed Particles (PA) design under tensile loading conditions.

File Name: Supplementary Movie 13

Description: The results of the dynamic explicit FEM simulations of the Gyroid + Particles (GP) design under tensile loading conditions.

File Name: Supplementary Movie 14

Description: The results of the dynamic explicit FEM simulations of the Collagen-like helices (OC) design under tensile loading conditions.

File Name: Supplementary Movie 15

Description: The results of the photopolymer blending study, where a Gradientless (ctrl) design and no blending effects are considered.

File Name: Supplementary Movie 16

Description: The results of the photopolymer blending study, where a Gradientless (ctrl) design and blending effects are considered.

File Name: Supplementary Movie 17

Description: The results of the photopolymer blending study, where a randomly distributed Particles (PA) design and no blending effects are considered.

File Name: Supplementary Movie 18

Description: The results of the photopolymer blending study, where a randomly distributed Particles (PA) design and blending effects are considered.
